# Characterizing the Crucial Components of Iron Homeostasis in the Maize Mutants *ys1* and *ys3*

**DOI:** 10.1371/journal.pone.0062567

**Published:** 2013-05-08

**Authors:** Tomoko Nozoye, Hiromi Nakanishi, Naoko K. Nishizawa

**Affiliations:** 1 Department of Global Agricultural Sciences, Graduate School of Agricultural and Life Sciences, The University of Tokyo, Tokyo, Japan; 2 Research Institute for Bioresources and Biotechnology, Ishikawa Prefectural University, Nonoichi Ishikawa, Japan; United States Department of Agriculture, Agricultural Research Service, United States of America

## Abstract

To acquire iron (Fe), graminaceous plants secrete mugineic acid family phytosiderophores through the phytosiderophore efflux transporter TOM1 and take up Fe in the form of Fe(III)–phytosiderophore complexes. *Yellow stripe 1* (*ys1*) and *ys3* are recessive mutants of maize (*Zea mays* L.) that show typical symptoms of Fe deficiency, i.e., interveinal chlorosis of the leaves. The *ys1* mutant is defective in the Fe(III)–phytosiderophore transporter YS1 and is therefore unable to take up Fe(III)–phytosiderophore complexes. While the *ys3* mutant has been shown to be defective in phytosiderophores release, the causative gene has not been identified. The present study was performed to characterize the expression profiles of the genes in *ys1* and *ys3* mutants to extend our understanding of Fe homeostasis in maize. Using quantitative real-time polymerase chain reaction, we assessed changes in the levels of gene expression in response to Fe deficiency of genes involved in Fe homeostasis, such as those related to phytosiderophore biosynthesis and Fe transport. As with other crops, these Fe deficiency-inducible genes were also upregulated in maize. In addition, these Fe deficiency-inducible genes were upregulated in both the *ys1* and *ys3* mutants, even under Fe-sufficient conditions. Indeed, the Fe concentrations in the roots of *ys1* and *ys3* plants were lower than that of wild-type controls. These results suggest that *ys1* and *ys3* are Fe-deficient during growth in the presence of Fe. In agreement with previous reports, the level of *YS1* expression decreased in the *ys1* mutant. Moreover, the expression level of a homolog of *TOM1* in maize decreased significantly in the *ys3* mutant. Unspliced introns of *ZmTOM1* were detected only in *ys3*, and not in *YS1YS3* or *ys1*, suggesting that *ZmTOM1* may be involved in the *ys3* phenotype.

## Introduction

Iron (Fe) is an essential nutrient for virtually all living organisms. Fe plays a key role in electron transfer in both photosynthetic and respiratory reactions in chloroplasts and mitochondria. Although abundant in mineral soils, Fe is sparingly soluble under aerobic conditions at high soil pH and exists mainly as oxidized insoluble Fe(III) compounds. Consequently, plants grown on calcareous soils often exhibit severe chlorosis due to Fe deficiency, which results in reduced crop yields [1].

Higher plants have two strategies for the uptake of Fe(III) from the rhizosphere [2]. All higher plants, with the exception of graminaceous plants, take up Fe using ferric-chelate reductases (FROs) to reduce Fe(III) to Fe(II), which is subsequently taken up by ferrous Fe transporters (IRTs; Strategy I; [3]–[5]). Alternatively, graminaceous plants secrete Fe chelators called mugineic acid family phytosiderophores (MAs) from their roots via Transporter Of MAs (TOM1) to solubilize Fe in the rhizosphere (Strategy II; [6], [7]). Graminaceous plants then take up Fe as Fe(III)–MAs complexes from the rhizosphere through the action of Yellow Stripe 1 (YS1) transporters at the plasma membrane [8], [9].

The biosynthetic pathway for MAs in graminaceous plants has been elucidated [7], [10]–[13]. Methionine, which is a precursor of MAs [10], is converted to 2′-deoxymugineic acid (DMA) via a series of reactions. While maize (*Zea mays* L.) and rice (*Oryza sativa* L.) secrete DMA, other species, including barley (*Hordeum vulgare* L.) and rye (*Secale cereale* L.), further hydroxylate DMA to other MAs. The genes that encode the biosynthetic enzymes responsible for converting *S*-adenosyl methionine to MAs have been identified in barley (*HvNAS1-7*, *NASHOR1* and *2*, *HvNAAT-A* and *-B*, *HvDMAS1*; [14]–[18]), maize (*ZmNAS1-3*, *ZmNAAT1*, *ZmDMAS1* [14], [19], [20]), and rice (*OsNAS1-3*, *OsNAAT1*, *OsDMAS1* [14], [20]–[22]). Expression of these genes is strongly induced by Fe deficiency. The non-proteinogenic amino acid nicotianamine (NA), which serves as an intermediate in the MAs biosynthetic pathway, also functions as a transition metal chelator in plants [23]–[25].

TOM1, which is a major facilitator superfamily (MFS) antiporter, was identified as an efflux transporter of DMA in rice [26]. *TOM1* expression is strongly induced in Fe-deficient roots. DMA secretion from rice roots is increased by the overexpression of *TOM1* and decreased by its repression, indicating that *TOM1* encodes the efflux transporter of DMA in plants [26].

Maize belongs to the C_4_ grasses; it has high photosynthetic efficiency and is important not only as a staple crop but also for adaptation to the environment. Under Fe-deficient conditions, as in calcareous soils, maize is highly susceptible to Fe deficiency and its growth decreases dramatically. However, as little information is available regarding genes in maize compared to other crops, such as rice, the molecular components involved in Fe homeostasis are not well understood. Two maize mutants, *yellow stripe-1* (*ys1*) and *yellow stripe 3* (*ys3*), are considered to be defective in Fe uptake mainly because they exhibit typical Fe-deficiency chlorosis (yellowing between the veins), which can be rescued by the administration of ferric chelates to the leaves [27]–[29]. The *ys1* mutant has been shown to be defective in the uptake of Fe(III)–DMA complexes [30]. The causative gene of the *ys1* mutant is *YS1*, which encodes the specific transporter responsible for the uptake of Fe-chelated DMA complexes from the rhizosphere into root cells [9]. While *YS1* expression increases in both roots and shoots under conditions of Fe deficiency, it is not regulated by zinc (Zn) or copper (Cu) deficiency [9], [31]. YS1 functions as a proton-coupled symporter for various DMA-bound metals, including Fe(III), Zn(II), Cu(II), and nickel (Ni)(II) [32]. YS1 also transports NA-chelated Ni(II), Fe(II), and Fe(III) complexes. Eighteen *YS1*-like (YSL) genes have been identified in rice [33]. Among these, *OsYSL15* encodes an Fe(III)–DMA transporter that appears to be an ortholog of *YS1* [34], [35]. OsYSL18 and OsYSL16 also transport Fe(III)–DMA [36], [37]. OsYSL2 transports Fe(II)–NA and manganese (Mn)(II)–NA, but not Fe(III)–DMA, and has been suggested to be responsible for phloem transport of Fe and Mn [33], [38]. Non-graminaceous plants also possess *YSL* genes, which encode transporters that are considered to play important roles in internal metal homeostasis by transporting metal–NA complexes, as non-graminaceous plants synthesize NA but not MAs [39]–[43]. In contrast, the *ys3* mutant has been reported to show impaired DMA secretion [44], although the causative gene has not yet been identified.

In the present study, quantitative real-time polymerase chain reaction (PCR) was performed to determine the regulation of genes involved in Fe homeostasis in maize. First, we confirmed that the expression of genes homologous to those involved in Fe homeostasis in rice were induced in maize under Fe-deficient conditions. The expression levels of the genes putatively involved in Fe homeostasis, such as the MAs biosynthetic pathway, as well as those for transcription factors and transporters, were induced in maize under Fe-deficient conditions. Second, the gene expression profiles of the *ys1* and *ys3* mutants were analyzed. The expression levels of the Fe-inducible genes were higher in both *ys1* and *ys3* compared to *YS1, YS3* [wild type (WT), i.e., (YS3WT), which is the same cultivar and has the same genetic background as the *ys3* mutant] even under Fe-sufficient conditions. These results suggest that *ys1* and *ys3* experience Fe deficiency even under Fe-sufficient conditions. In agreement with previous reports, the expression level of *YS1* in *ys1* decreased in comparison to the WT. Moreover, the expression level of the *TOM1* homolog (*ZmTOM1*) in *ys3* decreased compared to the WT or *ys1*. Unspliced *TOM1* mRNAs were detected only in *ys3*. *ZmTOM1* is located in the quantitative trait locus (QTL) of the *ys3* phenotype, and our results suggested that *ZmTOM1* may be involved in the *ys3* phenotype.

## Results

### Phenotyping of *ys1* and *ys3*

**Figure 1:**
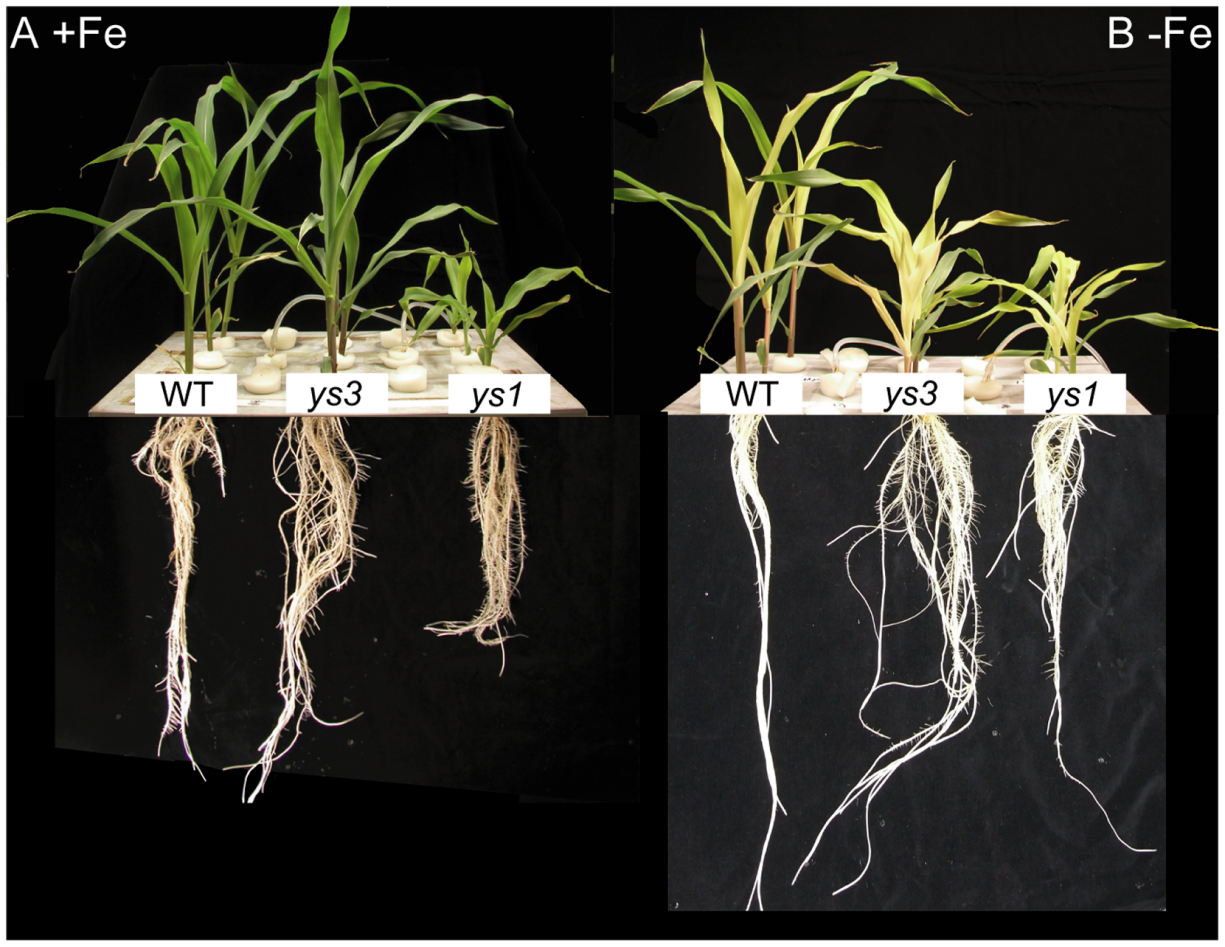
Maize plants used in the analysis. Maize seeds were germinated for 4 days on filter paper and then grown hydroponically for 17 days. (A) Fe-sufficient conditions. Twelve days after germination, the plants were transferred to Fe-free culture medium for 5 days. (B) Fe-deficient conditions.

**Figure 2:**
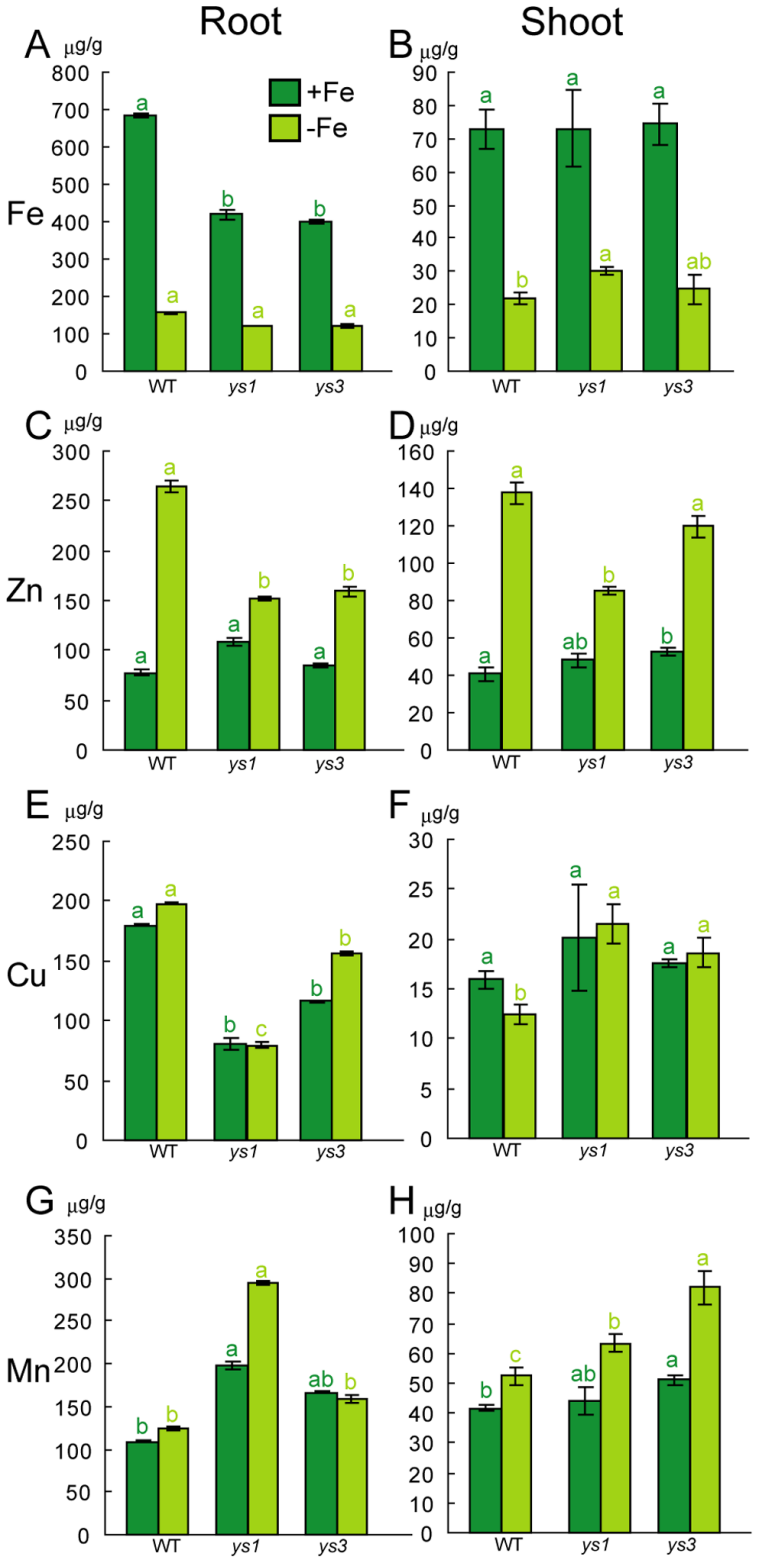
Metal determination of the plants. Concentrations of iron (Fe; A, B), zinc (Zn; C, D), copper (Cu; E, F), and manganese (Mn; G, H) in the roots and shoots of the WT and *ys1* and *ys3* plants were analyzed by inductively coupled plasma atomic emission spectrometry. +Fe, Fe-sufficient conditions; –Fe, Fe-deficient conditions. The values in the bars followed by different letters differ significantly from each other according to the Tukey-Kramer HSD test (*n* = 3, *P*<0.05).

Maize plants grown hydroponically under Fe-sufficient or Fe-deficient conditions for 5 days (Figure 1) were harvested at the same time. WT plants and the *ys1* and *ys3* mutants were grown together in a 20-L plastic container. The cultivar backgrounds used for the WT and the *ys1* and *ys3* mutants differed from each other. Thus, while comparing the mutants, note that the differences in phenotype are the cumulative effects of the *ys1* and *ys3* mutations in addition to the genetic differences between these cultivars. Under Fe-sufficient conditions, the *ys3* mutants showed slightly better growth than the WT, while root and shoot lengths were significantly shorter in the *ys1* mutant (Figure 1A). Under Fe-deficient conditions, shoot length decreased significantly in the *ys1* and *ys3* mutants, although no significant difference in root length was noted for these two mutants (Figure 1B). In the roots, the concentrations of Fe in the *ys1* and *ys3* mutants were lower than those in the WT plants when grown under control conditions (Figure 2A). No significant differences were observed in root Fe concentrations when plants were grown under Fe-deficient conditions (Figure 2A). In the shoot tissues, the Fe concentration was higher in the *ys1* plants than in the WT and *ys3* plants when grown under Fe-deficient conditions, while no significant differences in shoot Fe concentration were noted under Fe-sufficient conditions (Figure 2B). In addition to Fe, significant differences in the concentrations of other metals were also detected. In the roots, the concentration of Zn was significantly lower in the *ys1* and *ys3* mutants than in the WT plants under Fe-deficient conditions (Figure 2C). In the shoot tissues, the *ys1* and *ys3* plants showed higher Zn concentrations under Fe-sufficient conditions, while the Zn concentration was significantly lower in the *ys1* mutant than in the WT and *ys3* plants under Fe-deficient conditions (Figure 2D). In the roots, the *ys1* and *ys3* mutants showed significantly lower Cu concentrations under both Fe-sufficient and Fe-deficient conditions (Figure 2E). In shoot tissues, no significant differences were observed in the Cu concentrations when the plants were grown under Fe-sufficient conditions, while the *ys1* and *ys3* shoots showed higher Cu concentrations under Fe-deficient conditions (Figure 2F). In the roots, the concentration of Mn in the *ys1* mutants was higher than in the WT under both Fe-sufficient and Fe-deficient conditions (Figure 2G). When the plants were grown under Fe-sufficient conditions, the Mn concentration in the *ys3* mutants was slightly higher than those in WT and *ys1* plants, while the Mn concentrations in the *ys1* and *ys3* mutants were higher in the shoots under Fe-deficient conditions (Figure 2H). These results clearly indicate that metal homeostasis is significantly impaired in *ys1* and *ys3* mutants.

### Changes in the Expression of Fe Homeostasis-related Genes

**Figure 3:**
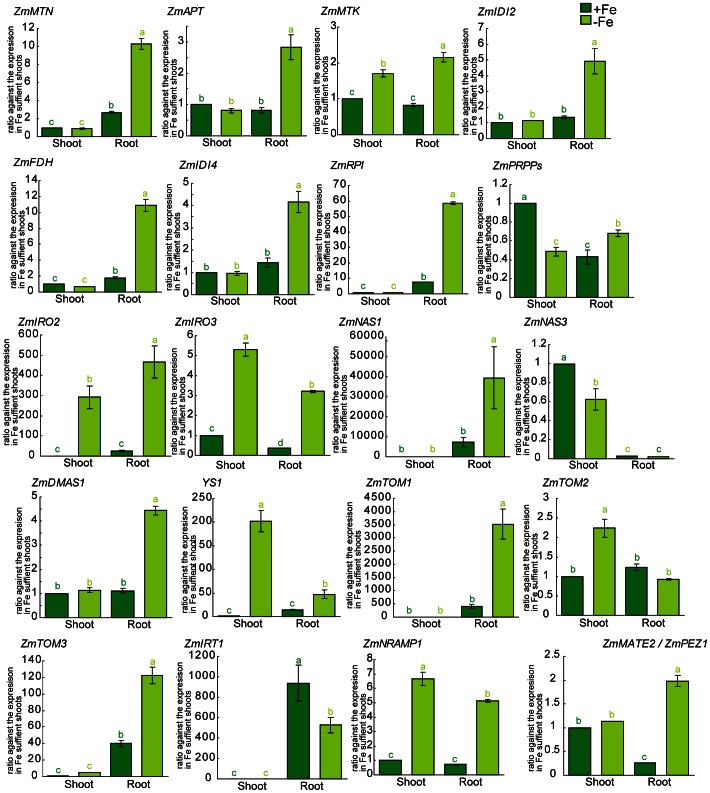
Expression change of maize homologs of genes involved in Fe homeostasis in rice. Quantitative real-time PCR of homologs of genes involved in the methionine cycle (*ZmMTN*, GRMZM2G171111; *ZmAPT*, GRMZM2G093347; *ZmMTK*, GRMZM2G464137; *ZmIDI2*, GRMZM2G139533; *ZmFDH*, GRMZM2G049811; *ZmIDI4*, GRMZM2G067265; *ZmRPI*, GRMZM2G035599; *ZmPRPPs*, GRMZM2G065030), transcription (*ZmIRO2*, GRMZM2G057413; *ZmIRO3*, GRMZM2G350312), MAs biosynthesis (*ZmNAS1*, GRMZM2G034956; *ZmNAS3*, GRMZM2G478568; *ZmDMAS1*, GRMZM2G060952), and transport (*ZmYS1*, GRMZM2G156599; *ZmTOM1*, GRMAM2G063306; *ZmTOM2*, GRMZM5G877788; *ZmTOM3*, GRMZM2G141081; *ZmIRT1*, GRMZM2G118821; *ZmNRAMP1*, GRMZM2G178190; *ZmMATE2/ZmPEZ1*, GRMZM2G170128) was performed with appropriate primers (Table S1 in File S1). These data are shown as ratios relative to the expression in Fe-sufficient shoots. The ubiquitin gene (UBQ) was used to normalize data. S.D. was calculated from experimental replicates (*n* = 3). Column bars followed by different letters are significantly different from each other according to the Tukey-Kramer HSD test (*n* = 3, *P*<0.05). Biological replicates were confirmed by repeating the experiment (Figure S1 in File S1). +Fe, Fe-sufficient conditions; –Fe, Fe-deficient conditions.

In Fe-deficient rice, the genes that encode the enzymes involved in the MAs biosynthetic pathway, including the methionine cycle, as well as those for transcription factors and transporters involved in Fe homeostasis, are induced, and their functions have been characterized [14], [24], [26], [34], [45]–[49]. Therefore, the differences in expression levels of the maize homologs of these genes between Fe-sufficient and Fe-deficient conditions were examined by quantitative real-time PCR. The expression levels of maize homologs that participate in the methionine cycle (*ZmMTN*, GRMZM2G171111; *ZmAPT*, GRMZM2G093347; *ZmMTK*, GRMZM2G464137; *ZmIDI2*, GRMZM2G139533; *ZmFDH*, GRMZM2G049811; *ZmIDI4*, GRMZM2G067265; *ZmRPI*, GRMZM2G035599) and MAs biosynthesis (*ZmNAS1*, GRMZM2G034956; *ZmDMAS1*, GRMZM2G060952) were markedly elevated in roots under Fe-deficient conditions (Figure 3). The expression level of *ZmNAS3* was higher in the shoots than the roots, and its expression decreased under Fe-deficient conditions (Figure 3; *ZmNAS3*, GRMZM2G478568). The expression levels of the homologs of the *OsIRO2* and *OsIRO3* genes (*ZmIRO2*, GRMZM2G057413; *ZmIRO3*, GRMZM2G350312), which are Fe deficiency-induced transcription factors that regulate the Fe-deficiency response [45]–[49], were markedly elevated in both roots and shoots under Fe-deficient conditions (Figure 3). *YS1* was also induced in both roots and shoots under Fe-deficient conditions (Figure 3; *YS1*, GRMZM2G156599). Transporter genes with similarities to *TOM1* and *TOM3* were induced in the roots under Fe-deficient conditions (Figure 3; *ZmTOM1*, GRMAM2G063306; *ZmTOM3*, GRMZM2G141081). In contrast, the *TOM2* homolog was induced only in the shoots, and not in the roots, under Fe-deficient conditions (Figure 3; *ZmTOM2*, GRMZM5G877788). The expression levels of the *OsIRT1*-homolog, which is involved in the transport of ferrous Fe [50], [51], were not induced in either the roots or shoots by Fe deficiency (Figure 3; *ZmIRT1*, GRMZM2G118821). Among the *NRAMP* family members, which transport various divalent metals, including Fe, Mn, and cadmium [52]–[54], the expression levels of *OsNRMAP2* homologs increased in both roots and shoots under Fe-deficient conditions (Figure 3; *ZmNRAMP1*, GRMZM2G178190). The expression levels of the phenolics efflux zero *PEZ* [55]–[57] homologs increased strongly in both roots and shoots under Fe-deficient conditions (Figure 3; *ZmMATE2*/*ZmPEZ1*, GRMZM2G170128).

### Gene Expression Profiles of *ys1* and *ys3* Mutants

**Figure 4:**
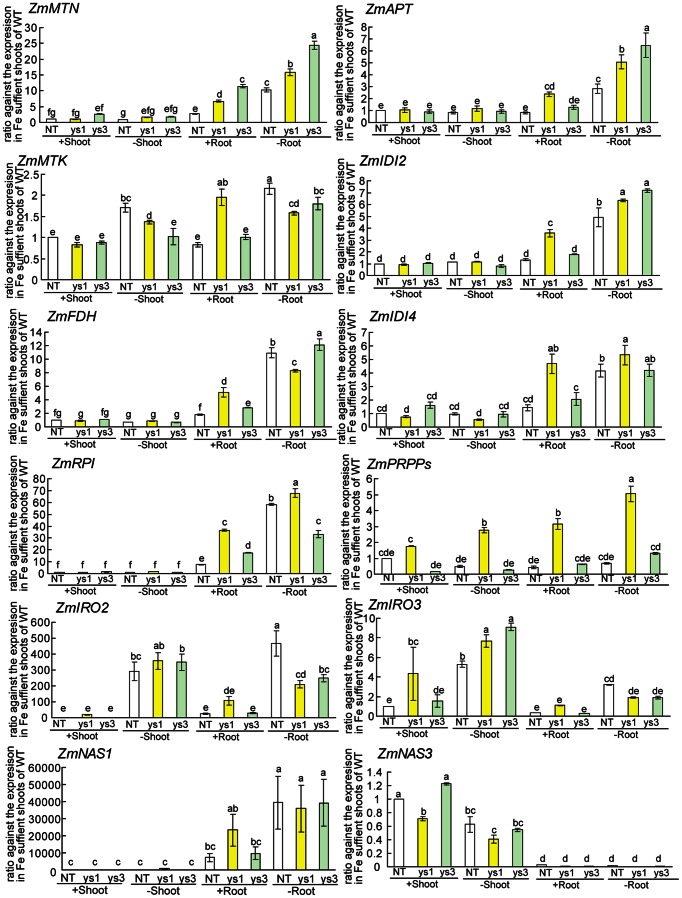
Differences in expression levels of Fe deficiency-inducible genes among WT, *ys1*, and *ys3*. The differences in gene expression among the nonmutant [wild-type (WT) cv. Alice] and *ys1* or *ys3* mutants were analyzed by quantitative real-time PCR. Quantitative real-time PCR of the genes involved in the methionine cycle (*ZmMTN*, GRMZM2G171111; *ZmAPT*, GRMZM2G093347; *ZmMTK*, GRMZM2G464137; *ZmIDI2*, GRMZM2G139533; *ZmFDH*, GRMZM2G049811; *ZmIDI4*, GRMZM2G067265; *ZmRPI*, GRMZM2G035599; *ZmPRPPs*, GRMZM2G065030), transcription (*ZmIRO2*, GRMZM2G057413; *ZmIRO3*, GRMZM2G350312), and MAs biosynthesis (*ZmNAS1*, GRMZM2G034956; *ZmNAS3*, GRMZM2G478568) was performed with appropriate primers (Table S1 in File S1). The data are shown as ratios relative to the expression in Fe-sufficient WT shoots. The ubiquitin gene (UBQ) was used to normalize data. S.D. was calculated from experimental replicates (*n* = 3). Column bars followed by different letters are significantly different from each other according to the Tukey-Kramer HSD test (*n* = 3, *P*<0.05). Biological replicates were confirmed by repeating the experiment (Figure S2 in File S1). +, Fe-sufficient conditions; –, Fe-deficient conditions.

**Figure 5:**
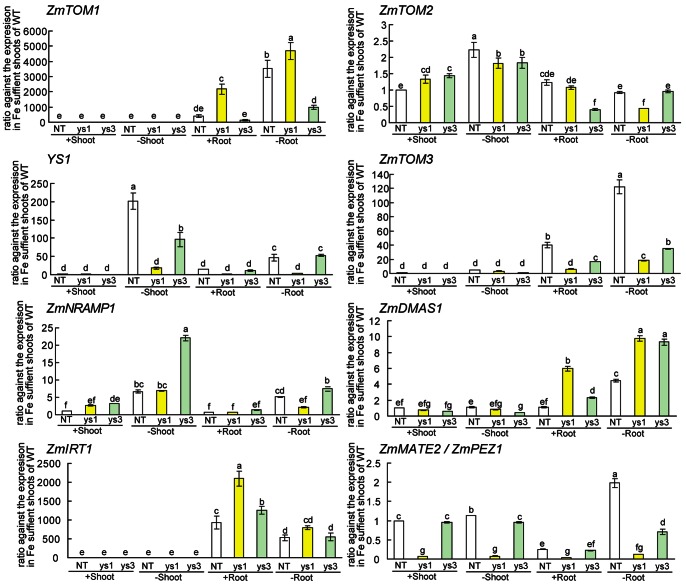
Differences in expression levels of Fe deficiency-inducible genes among WT, *ys1*, and *ys3*. The differences in gene expression among the nonmutant [wild-type (WT) cv. Alice] and *ys1* or *ys3* mutants were analyzed by quantitative real-time PCR. Quantitative real-time PCR of the genes involved in MAs biosynthesis (*ZmDMAS1*, GRMZM2G060952) and transport (*ZmYS1*, GRMZM2G156599; *ZmTOM1*, GRMAM2G063306; *ZmTOM2*, GRMZM5G877788; *ZmTOM3*, GRMZM2G141081; *ZmIRT1*, GRMZM2G118821; *ZmNRAMP1*, GRMZM2G178190; *ZmMATE2*/ZmPEZ1, GRMZM2G170128) was performed with appropriate primers (Table S1 in File S1). The data are shown as ratios relative to the expression in Fe-sufficient WT shoots. The ubiquitin gene (UBQ) was used to normalize data. S.D. was calculated from experimental replicates (*n* = 3). Column bars followed by different letters are significantly different from each other according to the Tukey-Kramer HSD test (*n* = 3, *P*<0.05). Biological replicates were confirmed by repeating the experiment (Figure S3 in File S1). +, Fe-sufficient conditions; –, Fe-deficient conditions.

The *ys1* and *ys3* mutants exhibit the Fe-deficient phenotype. The *ys1* mutant was reported to have a defect in the *YS1* gene and is unable to take up Fe–DMA from the soil [9], [30], while the *ys3* mutant was reported to have a defect in the secretion of DMA from the roots, although the causative gene has not been identified [43]. Changes in the expression levels of genes involved in biosynthesis and transport of MAs were examined in *ys1* and *ys3* (Figures 4, 5). As the background used in the present study differed for the WT and *ys1* and *ys3*, the sizes and sequences of the cDNA products for each gene were checked and confirmed to be the same. In addition, many of the genes linked to the methionine cycle were upregulated in *ys1* and *ys3*, especially under Fe-sufficient conditions (Figure 4; *ZmMTN*, *ZmAPT*, *ZmMTK*, *ZmIDI2*, *ZmFDH*, *ZmIDI4*, *ZmRPI*, *ZmPRPP*s). The expression levels of genes encoding NA synthase (*ZmNAS1*) and DMA synthase (*ZmDMAS1*) were also higher in the Fe-sufficient roots of *ys1* and *ys3* than in the WT (Figures 4, 5). In contrast, the expression level of *ZmNAS3* decreased in the shoots of the *ys1* mutant under both Fe-sufficient and Fe-deficient conditions (Figure 4), while it increased in *ys3* Fe-sufficient shoots. The expression levels of the *ZmIRO3* were also higher in the Fe-sufficient roots of *ys1* compared to the WT (Figure 4). The expression level of *ZmIRO2* decreased in both *ys1* and *ys3* (Figure 4). As reported previously, the expression level of *YS1* markedly decreased in *ys1*, although it was not different in *ys3* compared to the WT (Figure 5). The expression levels of *ZmNRAMP2* in Fe-sufficient shoots were higher in *ys1* and *ys3* than in the WT (Figure 5). The expression levels of *ZmIRT1* in Fe-sufficient and Fe-deficient roots were higher in *ys1* and *ys3* than in the WT (Figure 5). The expression level of *ZmMATE2* (homolog of *PEZ1*, *ZmPEZ1*) was lower in *ys1* compared to the WT and *ys3*.

**Figure 6:**
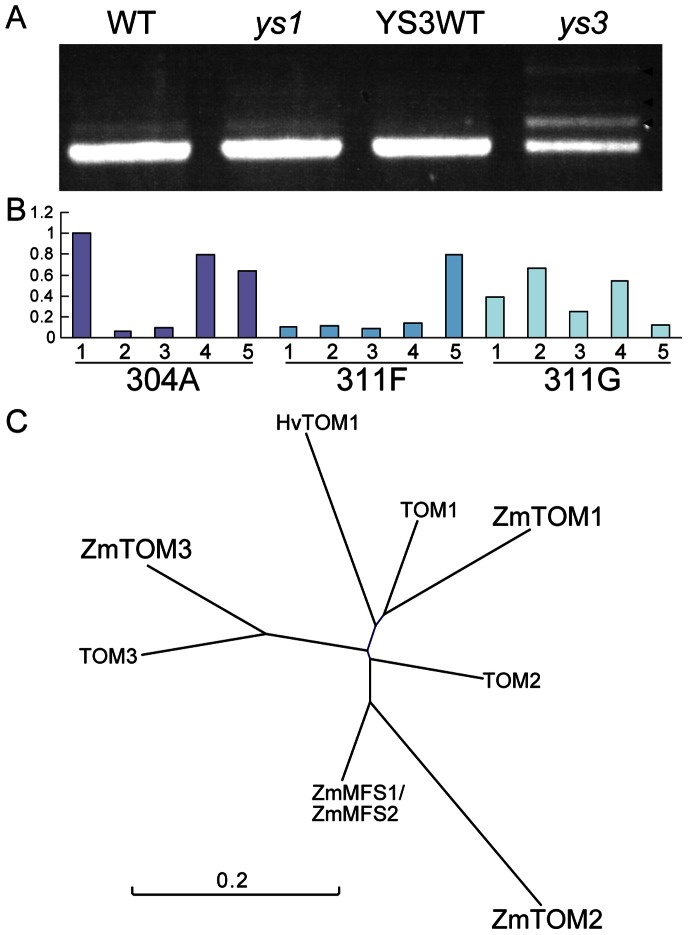
TOM1 family in maize. (A) Semiquantitative reverse transcription (RT)-PCR analysis of *ZmTOM1* (GRMZM2G063306_T02) in Fe-deficient roots of *ys1*, *ys3*, the WT, and YS3WT. Arrowheads represent the three bands only detected in *ys3*. (B) Quantitative real-time PCR of *ZmTOM1* in Fe-deficient roots of three *ys3* mutant lines (304A, 311F, and 311G). The ubiquitin gene (UBQ) was used to normalize data. These data are shown as ratios relative to the expression in 304A line #1. Five plants in each line were analyzed. The seeds were all from test crosses and should show 1∶1 segregation. (C) Phylogenetic tree of the TOM family.

As noted in the previous section, *ys3* was reported to have a defect in DMA secretion. In rice roots, the efficiency of DMA secretion was diminished by the repression of *TOM1*. The *ys3* mutants and *TOM1* repression rice both showed similar DMA secretion profiles, and the level secretion was reduced relative to the WT. Therefore, we analyzed the expression of homologs of *TOM1* (*ZmTOM1*, GRMAM2G063306; *ZmTOM2*, GRMZM5G877788; *ZmTOM3*, GRMZM2G141081), the efflux transporter of DMA by quantitative real-time PCR (Figure 5). The expression of *ZmTOM1* was mainly observed in the roots and was very low in the shoots in the WT and both *ys1* and *ys3* mutants. In roots, the expression of *ZmTOM1* in *ys3* was quite low compared to the WT and *ys1* under both Fe-sufficient and Fe-deficient conditions. The expression level of *ZmTOM3* was also high in the roots. The levels of *ZmTOM3* expression were lower in both *ys1* and *ys3* compared to the WT. In contrast, the expression level of *ZmTOM2* was higher in Fe-deficient shoots of *ys1*, *ys3*, and the WT, and no significant differences were observed among them. According to the Maize Genetics and Genomics Database (MaizeGDB), the *ys3* mutation is located within the interval of 85,750,522–114,783,939 on chromosome 3 (http://maizegdb.org/cgi-bin/locus_lookup_refgenv2.cgi?locus=ys3&id=IBM2); *ZmTOM1* (GRMZM2G063306) is also found within this interval (chromosome 3; 112,042,104–112,047,482). Therefore, we also confirmed the differences in size and sequence of the *ZmTOM1* cDNA between the WT and *ys3* by semiquantitative reverse transcription (RT)-PCR analysis (Figure 6). Consistent with quantitative real-time PCR analysis, the expression of *ZmTOM1* was lower in *ys3* than in the WT and *ys1* in Fe-deficient roots (Figure 6A). In *ys3*, three additional bands were detected in addition to the band that was of the same size as the *ZmTOM1* cDNA. The cultivars used for the WT and the *ys1* and *ys3* mutants differed from each other in the above experiment, and the difference in genetic background between WT and the *ys1* and *ys3* mutants may have affected *ZmTOM1* expression. Therefore, we examined *ZmTOM1* expression in YS3WT, which is the same cultivar and has the same genetic background as the *ys3* mutant. The expression of *ZmTOM1* was also induced in YS3WT under Fe-deficient conditions (Figure S3 in File S1). In Fe-deficient roots, the expression of *ZmTOM1* was higher in YS3WT than in *ys3*. The expression levels of other genes, such as *ZmTOM2* and *ZmTOM3*, were not different in *ys3* compared to YS3WT. The larger bands observed in *ys3* were not detected in YS3WT (Figure 6A). In addition, we cultivated an additional three *ys3* mutant lines (304A, 311F, and 311G) provided by the Maize Stock Center (http://www.maizegdb.org/cgi-bin/displayvarrecord.cgi?id=15373). As the seeds were all from test crosses, they should show 1∶1 segregation. We extracted mRNA from the Fe-deficient roots of five plants of each line, and *ZmTOM1* expression was analyzed by quantitative real-time PCR (Figure 6B). In all three lines, the progeny was segregated into plants in which the expression level of *ZmTOM1* was comparable to the WT or significantly lower than the WT. Furthermore, we sequenced the PCR products of *ZmTOM1* (GRMAM2G0633306_T02) in the WT and the *ys1* and *ys3* mutants. The larger bands observed only in *ys3* contained unspliced introns of *ZmTOM1.* Three patterns of intron insertions were noted (Figures S4, S5, S6 in File S1). These results suggest that *ZmTOM1* is involved in the phenotype of *ys3*.

## Discussion

### Homologs of Genes known to be Involved in Fe Homeostasis in Rice are also Induced by Fe Deficiency in Maize

Under Fe-deficient conditions, plants experience Fe-deficiency signals that trigger cellular activities to acquire and transport Fe into the plant body. In agreement with the observations in rice, the expression levels of genes homologous to those that participate in the methionine cycle (*ZmMTN*, GRMZM2G171111; *ZmAPT*, GRMZM2G093347; *ZmMTK*, GRMZM2G464137; *ZmIDI2*, GRMZM2G139533; *ZmFDH*, GRMZM2G049811; *ZmIDI4*, GRMZM2G067265; *ZmRPI*, GRMZM2G035599), MAs biosynthesis (*ZmNAS1*, GRMZM2G034956; *ZmDMAS1*, GRMZM2G060952), transcription (*ZmIRO2*, GRMZM2G057413; *ZmIRO3*, GRMZM2G350312), and Fe transport (*YS1*, GRMZM2G156599; *ZmTOM1*, GRMAM2G063306; *ZmTOM3*, GRMZM2G141081; *ZmNRAMP1*, GRMZM2G178190; *ZmMATE2*/*ZmPEZ1*, GRMZM2G170128) in maize were increased by Fe deficiency (Figure 3). These results support the suggestion that Fe deficiency triggers similar responses in rice and maize. Moreover, the expression of the *OsIRT1* homolog was not strongly induced in maize by Fe deficiency. In rice, ferrous Fe was reported to be acquired by the *OsIRT1* transporter [50], [51], and the expression of *OsIRT1* is strongly induced in Fe-deficient roots. Maize is an upland plant, while rice grows under submerged conditions, where ferrous Fe is abundant. This difference suggests that maize acquires Fe mainly by chelation, while rice absorbs ferrous Fe via OsIRT1 in addition to chelation under Fe-deficient conditions.

### Expression Profiles of *ys1* and *ys3*

The expression levels of genes involved in the methionine cycle (*ZmMTN*, GRMZM2G171111; *ZmAPT*, GRMZM2G093347; *ZmMTK*, GRMZM2G464137; *ZmIDI2*, GRMZM2G139533; *ZmFDH*, GRMZM2G049811; *ZmIDI4*, GRMZM2G067265; *ZmRPI*, GRMZM2G035599; *ZmPRPPs*, GRMZM2G065030) and MAs biosynthesis (*ZmNAS1*, GRMZM2G034956; *ZmDMAS1*, GRMZM2G060952) were higher in the Fe-sufficient roots of *ys1* and *ys3* plants than in those of the WT (Figures 4, 5). These results suggest that *ys1* and *ys3* are unable to acquire sufficient Fe in the roots, which triggers the Fe deficiency response, even under Fe-sufficient conditions. The expression levels of *ZmMTN*, *ZmAPT*, *ZmMTK*, *ZmIDI2*, *ZmFDH*, *ZmIDI4*, *ZmRPI*, *ZmPRPPs*, *ZmNAS1*, *ZmDMAS1*, *ZmIRO3*, and *TOM1* were higher in *ys1* than in *ys3* and the WT, suggesting that *ys1* senses Fe deficiency more strongly than *ys3*. This difference in gene expression may be linked to the observation that the shoots of *ys1* were much smaller than those of either the WT or *ys3* under Fe-sufficient conditions (Figure 1). Fe concentration and shoot biomass would suggest that *ys1* mutants are far less capable of Fe uptake than either *ys3* mutans or WT plants. The *ys1* mutant has been shown to have a mutation in *YS1* [9]. As described previously, the expression level of *YS1* had decreased in *ys1*. These observations confirm that *YS1* is defective in the *ys1* mutant and that this defect is responsible for the *ys1* phenotype. The YSL family has been suggested to be important not only for the acquisition of Fe(III)–DMA from the soil but also for the translocation of Fe from the root to shoot and seeds in rice [24], [33], [34], [36]–[38]. In addition, YS family genes were reported to be involved in the translocation of other metals, including Zn, Cu, and Mn [34], [35], [38], [41], [58]. In this study, *ys1* was smaller than the WT and *ys3* under both Fe-sufficient and Fe-deficient conditions (Figure 1). Moreover, in addition to Fe, the concentrations of other metals are altered in *ys1* mutants. These results suggest that *ys1* mutants suffer from Fe deficiency even under Fe-sufficient conditions and induce the expression of Fe deficiency-responsive genes.

In contrast to *ys1*, *ys3* showed no Fe-related visible phenotype under Fe-sufficient conditions. Instead, *ys3* showed slightly better growth than the WT. The Fe deficiency-inducible genes were slightly induced in *ys3* compared to the WT, but less than in *ys1* (Figures 4, 5). These results suggest that *ys3* suffers from Fe deficiency under Fe-sufficient conditions but not as strongly as *ys1*. In this experiment, *ys3* plants were grown beside the WT and *ys1* plants. *ys3* plants may have absorbed DMA secreted from the roots of WT and *ys1* plants, and grew better than *ys1*. In rice, *OsNRAMP1* and *OsNRAMP5* have been reported to play important roles in the absorption and translocation of ferrous Fe in rice [52]–[54]. In the present study, the maize plants were grown hydroponically where ferrous Fe was comparatively abundant. The expression level of the *OsNRAMP2* homolog (*ZmNRAMP1*; GRMZM2G178190) was upregulated in *ys3* as compared to the WT. The *ys3* mutant has been speculated to experience Fe deficiency in the roots as it cannot secrete DMA [44]. However, *ys3* could acquire ferrous Fe through NRAMP family transporters. As the level of DMA in the roots of *ys3* may have been high, Fe may have been efficiently transported from the roots to the shoots via YSL family members, perhaps because *ys3* has larger shoots than the WT.

### The Expression Level of DMA Efflux Transporter Decreased in *ys3*

The *ys3* mutant was reported to be defective in the secretion of DMA, although the causative gene has not been identified [44]. Recently, TOM1 was identified as a DMA efflux transporter in rice that mainly secretes DMA from the roots into the soil [26]. TOM1 and ENA1 belong to the major facilitator superfamily, and maize has many homologous genes (Figure S7 in File S1). In the present study, the expression level of the *TOM1* homolog (*ZmTOM1*, GRMZM2G063306_T02) decreased in *ys3* but not in *ys1* as compared to the WT and YS3WT. Similar to *TOM1*, *ZmTOM1* was mainly expressed in the roots and strongly induced by Fe deficiency. *ZmTOM1* is localized on chromosome 3; 112,042,104–112,047,482. In MaizeGDB, the *ys3* QTL is located on chromosome 3; 85,750,522–114,783,939 (http://maizegdb.org/cgi-bin/locus_lookup_refgenv2.cgi?locus=ys3&id=IBM2). Semiquantitative RT-PCR showed larger bands of this gene in *ys3*, but not in *ys1* or the WT (Figure 6). These larger bands were not observed in YS3WT, suggesting that the unspliced cDNA in *ys3* was not due to its genetic background. These observations suggest that *ZmTOM1* is responsible for the *ys3* phenotype. The larger bands corresponded to the unspliced mRNA of *TOM1* (Figures S4, S5, S6 in File S1). These results suggested that some mutation or insertion affect the splicing of *ZmTOM1* in the *ys3* mutant. The GRAMENE database shows that the genome has not been completely sequenced and part of the *ZmTOM1* genome information is missing (Figure S5 in File S1; http://www.gramene.org/Zea_mays/Gene/Sequence?g=GRMZM2G063306r=3:112042104-112047482). Further analysis is needed to determine why several patterns exist for the splicing of *ZmTOM1.*

In conclusion, using transcriptomic analyses, we identified the maize genes involved in the response to Fe deficiency. Furthermore, transcriptomic analyses revealed candidate genes for the *ys3* mutant. Further analysis may provide additional data to conclude that a defect in *ZmTOM1* is involved in the phenotype of the *ys3* mutant.

## Materials and Methods

### Plant Materials

The *ys1* and *ys3* mutant plants were grown from homozygous seeds. A WT cultivar (Alice) was used as a control, as even though it has a different genetic background from *ys1* and *ys3*, this line was previously used in a study of the *ys1* mutant [30]. To confirm the expression of *ZmTOM1*, another WT line (YS3WT) with the same genetic background as *ys3* was used [44]. Three additional lines of *ys3* mutants (304A, 311F, and 311G) were also analyzed.

### Hydroponic Culture

Seeds were germinated for 4 days in the dark at 25°C on filter paper soaked with distilled water. Seedlings were then grown hydroponically in a nutrient solution containing 0.7 mM K_2_SO_4_, 0.1 mM KCl, 0.1 mM KH_2_PO_4_, 2.0 mM Ca(NO_3_)_2_, 0.5 mM MgSO_4_, 10 µM H_3_BO_3_, 0.5 µM MnSO_4_, 0.2 µM CuSO_4_, 0.5 µM ZnSO_4_, 0.05 µM Na_2_MoO_4_, and 0.1 mM Fe(III)–EDTA. The pH of the nutrient solution was adjusted daily to 5.5 with 1 M HCl. Plants were grown in a 5-L plastic container for 4 days and then transferred to a 20-L plastic container with air-bubbling. Fe deficiency was initiated 8 days after germination by transfer of the plants to Fe(III)–EDTA-free culture medium. Maize plants grown hydroponically under Fe-sufficient or Fe-deficient conditions for 5 days were harvested at the same time.

### Metal Determination

The roots or shoots of dried maize plants grown hydroponically were ground, and samples of 50 mg were used for metal determination. The samples were digested in 2 ml of 13 M HNO_3_ (Wako Pure Chemical, Osaka, Japan) at 210°C for 20 min with MARS Xpress (CEM Corp., Matthews, NC). After digestion, the samples were diluted to a volume of 5 ml and analyzed by inductively coupled plasma atomic emission spectrometry (SPS1200VR; Seiko, Tokyo, Japan). All experiments were performed in triplicate.

### RNA Extraction

The maize plants grown hydroponically were immediately frozen in liquid nitrogen. Total RNA was extracted from the shoots and roots of three plants per treatment using an RNeasy Plant Kit (Qiagen, Hilden, Germany) in accordance with the manufacturer’s instructions. The yield and purity of the RNA were determined spectrophotometrically. To confirm the biological replicates, RNA was separately extracted from the shoots and roots of three to five plants per treatment.

### Quantitative Real-time PCR and Semiquantitative RT-PCR

Total RNA (3 µg) was treated with RNase-free DNase I (Takara, Kyoto, Japan) to remove contaminating genomic DNA. First-strand cDNA was synthesized using ReverTra Ace reverse transcriptase (Toyobo, Tokyo, Japan) by priming with oligo-d(T)_30_. For quantitative RT-PCR, a fragment was amplified by PCR in a StepOnePlus Real-Time PCR system (Applied Biosystems, Foster City, CA) with SYBR Green I and ExTaq™ Real-Time PCR Version (Takara) according to the manufacturers’ instructions. The template concentration was adjusted to 30 ng per reaction. The primers used for real-time PCR are described in Table S1 in File S1. The primers used as the internal control (*ZmUbiquitin*, GRMZM2G118637) in RT-PCR were as follows: *ZmUbiquitin* forward, 5′-GTTGAAGCTGCTGCTGTATCTGG’-3′ and *ZmUbiquitin* reverse, 5′-GCGGTCGCACGATAGTTTTG-3′. Normalization of quantitative real-time PCR was performed by the comparative Ct method calculation according to the manufacturer’s instructions (Applied Biosystems StepOnePlus™ Real-Time PCR system). The data show the relative increase or decrease of the gene expression level in each sample compared to the gene expression levels in Fe-sufficient shoots of the non-transformant (NT) in three experimental replicates and three to five biological replicates. The standard deviation of the nonmutant segregant plants (YS3WT) was also calculated from three biological replications. The sizes and sequences of the amplified fragments were confirmed by agarose gel electrophoresis and with an automated sequencer (3130 Genetic Analyzer; Applied Biosystems), respectively. Analysis of variance with the Tukey-Kramer HSD test was used to compare data. The statistical software package JMP9 (SAS Institute, Cary, NC) was used in all analyses. All methods and data were confirmed to follow the MIQE guidelines [59]. For semiquantitative RT-PCR, 30 ng of cDNA was used for each reaction. PCR was performed using the GeneAmp PCR system 9700 (Applied Biosystems).

### Supporting Information

The raw data for quantitative real-time PCR in this study have been deposited in GEO (Accession No. GSE44557; http://www.ncbi.nlm.nih.gov/geo/query/acc.cgi?acc=GSE44557). The nucleotide sequence data reported in this paper have been deposited in the GRAMENE databases under the accession numbers *ZmTOM1*, GRMAM2G063306; *ZmTOM2*, GRMZM5G877788; *ZmTOM3*, GRMZM2G141081; *ZmMTN*, GRMZM2G171111; *ZmAPT*, GRMZM2G093347; *ZmMTK*, GRMZM2G464137; *ZmIDI2*, GRMZM2G139533; *ZmFDH*, GRMZM2G049811; *ZmIDI4*, GRMZM2G067265; *ZmRPI*, GRMZM2G035599; *ZmPRPPs*, GRMZM2G065030, *ZmIRO2*, GRMZM2G057413; *ZmIRO3*, GRMZM2G350312; *ZmNAS1*, GRMZM2G034956; *ZmNAS3*, GRMZM2G478568; *ZmDMAS1*, GRMZM2G060952; *ZmYS1*, GRMZM2G156599; *ZmIRT1*, GRMZM2G118821; *ZmNRAMP1*, GRMZM2G178190; *ZmMATE2/ZmPEZ1*, GRMZM2G170128.

## Supporting Information

File S1 **Supporting Information. Figure S1. Biological replicates for quantitative real-time PCR for the expression changes in maize of genes homologous to those involved in Fe homeostasis in rice.** The expression changes in maize [wild type (YS3WT), which was the same cultivar and had the same genetic background as the *ys3* mutant], of genes homologous to those involved in Fe homeostasis in rice. Quantitative real-time PCR of the genes homologous to those involved in the methionine cycle (*ZmMTN*, GRMZM2G171111; *ZmAPT*, GRMZM2G093347; *ZmMTK*, GRMZM2G464137; *ZmIDI2*, GRMZM2G139533; *ZmFDH*, GRMZM2G049811; *ZmIDI4*, GRMZM2G067265; *ZmRPI*, GRMZM2G035599; *ZmPRPPs*, GRMZM2G065030), transcription (*ZmIRO2*, GRMZM2G057413; *ZmIRO3*, GRMZM2G350312), MAs biosynthesis (*ZmNAS1*, GRMZM2G034956; *ZmNAS3*, GRMZM2G478568; *ZmDMAS1*, GRMZM2G060952), and transport (*ZmYS1*, GRMZM2G156599; *ZmTOM1*, GRMAM2G063306; *ZmTOM2*, GRMZM5G877788; *ZmTOM3*, GRMZM2G141081; *ZmIRT1*, GRMZM2G118821; *ZmNRAMP1*, GRMZM2G178190; *ZmMATE2/ZmPEZ1*, GRMZM2G170128) was performed with appropriate primers (Table S1 in File S1). The data are shown as ratios relative to the expression in Fe-sufficient shoots. The ubiquitin gene (UBQ) was used to normalize data. S.D. was calculated from the biological replicates (*n* = 5). Column bars followed by different letters are significantly different from each other according to the Tukey-Kramer HSD test (*n* = 5, *P*<0.05). +Fe, Fe sufficient conditions; –Fe, Fe-deficient conditions. **Figure S2. Biological replicates for quantitative real-time PCR to determine the differences in expression levels of Fe deficiency-inducible genes among** ***YS1, YS3*** **[wild-type (YS3WT), which is the same cultivar and has the same genetic background as the** ***ys3*** **mutant] and** ***ys1*** **or** ***ys3*** **mutants.** Quantitative real-time PCR of genes involved in the methionine cycle (*ZmIDI2*, GRMZM2G139533; *ZmFDH*, GRMZM2G049811; *ZmIDI4*, GRMZM2G067265; *ZmRPI*, GRMZM2G035599), transcription (*ZmIRO2*, GRMZM2G057413; *ZmIRO3*, GRMZM2G350312), and MAs biosynthesis (*ZmNAS1*, GRMZM2G034956; *ZmNAS3*, GRMZM2G478568) was performed with appropriate primers (Table S1 in File S1). The data are shown as ratios relative to the expression in Fe-sufficient WT shoots. The ubiquitin gene (UBQ) was used to normalize data. S.D. was calculated from the biological replicates (*n* = 3–6). Column bars followed by different letters are significantly different from each other according to the Tukey-Kramer HSD test (*n* = 3–6, *P*<0.05). +Fe, Fe-sufficient conditions; –Fe, Fe-deficient conditions. **Figure S3. Biological replicates for quantitative real-time PCR to determine differences in the expression levels of Fe deficiency-inducible genes among** ***YS1,YS3*** **[wild-type (YS3WT), which is the same cultivar and has the same genetic background as the** ***ys3*** **mutant] and** ***ys1*** **or** ***ys3*** **mutants.** Quantitative real-time PCR of the genes involved in MAs biosynthesis (*ZmDMAS1*, GRMZM2G060952) and transport (*ZmYS1*, GRMZM2G156599; *ZmTOM1*, GRMAM2G063306; *ZmTOM2*, GRMZM5G877788; *ZmTOM3*, GRMZM2G141081; *ZmIRT1*, GRMZM2G118821; *ZmNRAMP1*, GRMZM2G178190; *ZmMATE2*/ZmPEZ1, GRMZM2G170128) was performed with appropriate primers (Table S1 in File S1). The data are shown as ratios relative to the expression in Fe-sufficient YS3WT shoots. The ubiquitin gene (UBQ) was used to normalize data. S.D. was calculated from the biological replicates (*n* = 3–6). Column bars followed by different letters are significantly different from each other according to the Tukey-Kramer HSD test (*n* = 3–6, *P*<0.05). +Fe, Fe-sufficient conditions; –Fe, Fe-deficient conditions. **Figure S4. Unspliced introns of** ***ZmTOM1*** **in** ***ys3*** **.** GRMZM2G063306_T02orf, *ZmTOM1* cDNA sequence predicted by GRAMENE; ZmTOM1pcr, partial sequence of *ZmTOM1* used for quantitative real-time PCR; ZmTOM1orf, sequenced *ZmTOM1* from the WT; Pattern_1, intron insertion version of *ZmTOM1* cDNA in *ys3*; Pattern_2, intron insertion version of *ZmTOM1* cDNA in *ys3*; Pattern_3, intron insertion version of *ZmTOM1* cDNA in *ys3*. **Figure S5. Genomic sequence of GRMZM2G063306.** Colored and bold font indicates the assembled plant exon in this region. Pink font shows the region where sequencing was not perfect. **Figure S6. Full-length cDNA of** ***ZmTOM1*** **.** GRMZM2G063306_T02orf, *ZmTOM1* cDNA sequence predicted by GRAMENE; ZmTOM1pcr, partial sequence of *ZmTOM1* used for quantitative real-time PCR; ZmTOM1orf, sequenced *ZmTOM1* from the WT. **Figure S7. Phylogenetic tree of** ***TOM1*** **and** ***ENA1*** **homologs in maize.** GRAMENE was searched for homologous genes of *ENA1* and *TOM1*. Scale bar, 0.3 substitutions/site. **Table S1.**
(PDF)
