# Supplementary material for: Characterizing the Crucial Components of Iron Homeostasis in the Maize Mutants ys1 and ys3
Source: PLoS One. 2013 May 8;8(5):e62567. doi: 10.1371/journal.pone.0062567 (PMC3648533; doi:10.1371/journal.pone.0062567)
Supplement: File S1 — Supporting Information. Figure S1. Biological replicates for quantitative real-time PCR for the expression changes in maize of genes homologous to those involved in Fe homeostasis in rice. The expression changes in maize [wild type (YS3WT), which was the same cultivar and had the same genetic background as the ys3 mutant], of genes homologous to those involved in Fe homeostasis in rice. Quantitative real-time PCR of the genes homologous to those involved in the methionine cycle (ZmMTN, GRMZM2G171111; ZmAPT, GRMZM2G093347; ZmMTK, GRMZM2G464137; ZmIDI2, GRMZM2G139533; ZmFDH, GRMZM2G049811; ZmIDI4, GRMZM2G067265; ZmRPI, GRMZM2G035599; ZmPRPPs, GRMZM2G065030), transcription (ZmIRO2, GRMZM2G057413; ZmIRO3, GRMZM2G350312), MAs biosynthesis (ZmNAS1, GRMZM2G034956; ZmNAS3, GRMZM2G478568; ZmDMAS1, GRMZM2G060952), and transport (ZmYS1, GRMZM2G156599; ZmTOM1, GRMAM2G063306; ZmTOM2, GRMZM5G877788; ZmTOM3, GRMZM2G141081; ZmIRT1, GRMZM2G118821; ZmNRAMP1, GRMZM2G178190; ZmMATE2/ZmPEZ1, GRMZM2G170128) was performed with appropriate primers (Table S1 in File S1). The data are shown as ratios relative to the expression in Fe-sufficient shoots. The ubiquitin gene (UBQ) was used to normalize data. S.D. was calculated from the biological replicates (n = 5). Column bars followed by different letters are significantly different from each other according to the Tukey-Kramer HSD test (n = 5, P<0.05). +Fe, Fe sufficient conditions; –Fe, Fe-deficient conditions. Figure S2. Biological replicates for quantitative real-time PCR to determine the differences in expression levels of Fe deficiency-inducible genes among YS1, YS3 [wild-type (YS3WT), which is the same cultivar and has the same genetic background as the ys3 mutant] and ys1 or ys3 mutants. Quantitative real-time PCR of genes involved in the methionine cycle (ZmIDI2, GRMZM2G139533; ZmFDH, GRMZM2G049811; ZmIDI4, GRMZM2G067265; ZmRPI, GRMZM2G035599), transcription (ZmIRO2, GRMZM2G057413; ZmIRO3, GRMZM2G350312), and MAs biosynthesis (ZmNAS1, [file pone.0062567.s001.pdf]

Table S1. Primers used for quantitative real-time PCR

gene name	gene ID in gramene	FW (3'-5')	RV (3'-5')
ZmMTN	GRMZM2G171111	GCTAGGCTACGAACAATACA	ACATCTGGATTAGGAGTAGG
ZmAPT	GRMZM2G093347	ACAAACTTGGGGACAGGCCA	ACTCTAACTCTCTGAGAAGA
ZmMTK	GRMZM2G464137	CCTGCCTGATATATACAACA	CTTGCTGGCATCCTTGATTG
ZmIDI2	GRMZM2G139533	CTCTTCATTCGGGGGGAGTT	GGAGAGATCAATGGAAGTTA
ZmFDH	GRMZM2G049811	CTGATTCTGCTCAGGAACTT	TCTCCGTAAGAGGTGTGTTG
ZmIDI4	GRMZM2G067265	CTGAGGCAATAGCTGCAACC	AGACCTCATCTGAGAACAGC
ZmRPI	GRMZM2G035599	GGCGTCGTCGAGCACGGCAT	CTTCCTTTCCATGACTGCGA
ZmPRPPs	GRMZM2G065030)	GACCTCCGTTTGAGGTTCTG	CGAGCATCGACAGTAAGTAC
ZmIRO2	GRMZM2G057413	AACGACCTCTACTCCTCGCT	CTGCAGCTCCGGGATGTACT
ZmIRO3	GRMZM2G350312	AGATTCATAAGGCTGAGAGG	TCGAAGAGAGTCTACTTGAA
ZmNAS1	GRMZM2G034956	ATCCTGAGGACATTCGTCGC	CAGCGATTTGTAACTATTAT
ZmNAS3	GRMZM2G478568	CGTGTCTACACCACATGCGT	GAGCTAAGCTACATGCTAA
ZmDMAS1	GRMZM2G060952	CTGATCGTGAAGAGCTTCGA	GCAGGGCAGTGGCACGCATT
YS1	GRMZM2G156599	CACTCATCAGCAAGCAGAAA	TGCAGACTCTTAACAGTGAC
ZmTOM1	GRMAM2G063306	AAGTGTAAATTCATGCCGTG	GATCCCCTGGAAAGAAGGCA
ZmTOM2	GRMZM5G877788	GTTTCGTCGGCGCTATCCAT	AAGAACGCGGCATGCTGGCG
ZmTOM3	GRMZM2G141081	GTGCTTTCAGTCACAGGCGT	ATAGGTCCAAGGATTTTGTT
ZmIRT1	GRMZM2G118821	CTCAGATACGAATACGATCA	TCCAGCTCCTCTGCTTCCTC
ZmNRAMP1	GRMZM2G178190	GAGCAAATCATGGGGTCGTT	TGGATCTACAGCACTGTTCC
ZmMATE2 / ZmPEZ1	GRMZM2G170128	CTCCTAGGCTTCTACTTTGA	AGTTGGTCCTGAATGTAACC

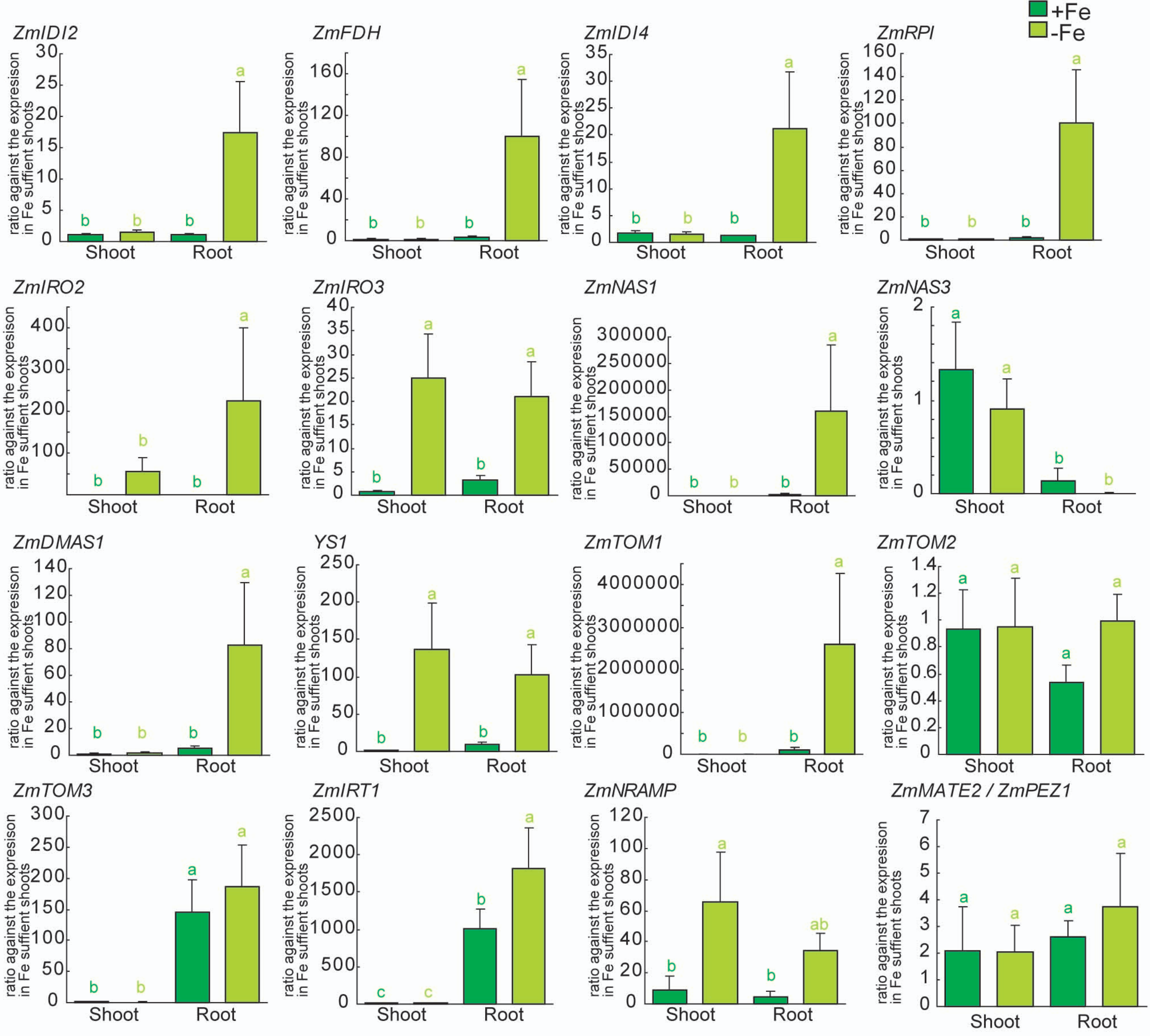

Figure S1. Biological replicates for quantitative real-time PCR for the expression changes in maize of the homologous genes involved in Fe homeostasis in rice. The expression change of the homolgous genes in maize (wild type (YS3WT), which is the same cultivar and has the same genetic background with *ys3* mutant) which is involved in Fe homeostasis in rice. The quantitative real-time PCR of the homologous genes which is involved in the methionine cycle (*ZmMTN*, GRMZM2G171111; *ZmAPT*, GRMZM2G093347; *ZmMTK*, GRMZM2G464137; *ZmIDI2*, GRMZM2G139533; *ZmFDH*, GRMZM2G049811; *ZmIDI4*, GRMZM2G067265; *ZmRPI*, GRMZM2G035599; *ZmPRPPs*, GRMZM2G065030), transcription (*ZmIRO2*, GRMZM2G057413; *ZmIRO3*, GRMZM2G350312), MAs biosynthesis (*ZmNAS1*, GRMZM2G034956; *ZmNAS3*, GRMZM2G478568; *ZmDMAS1*, GRMZM2G060952), and transport (*ZmYS1*, GRMZM2G156599; *ZmTOM1*, GRMAM2G063306; *ZmTOM2*, GRMZM5G877788; *ZmTOM3*, GRMZM2G141081; *ZmIRT1*, GRMZM2G118821; *ZmNRAMP1*, GRMZM2G178190; *ZmMATE2/ZmPEZ1*, GRMZM2G170128) was performed with appropriate primers (Table S1 in File S1). The data was shown as ratio against the expression in Fe sufficient shoots. The ubiquitin gene (UBQ) was used to normalize data. S.D. was calculated from the biological replicates (n=5). Column bars followed by different letters are significantly different from each other according to the Turkey-Kramer HSD test (n=5, P<0.05).+Fe, Fe sufficient; -Fe, Fe defficient.

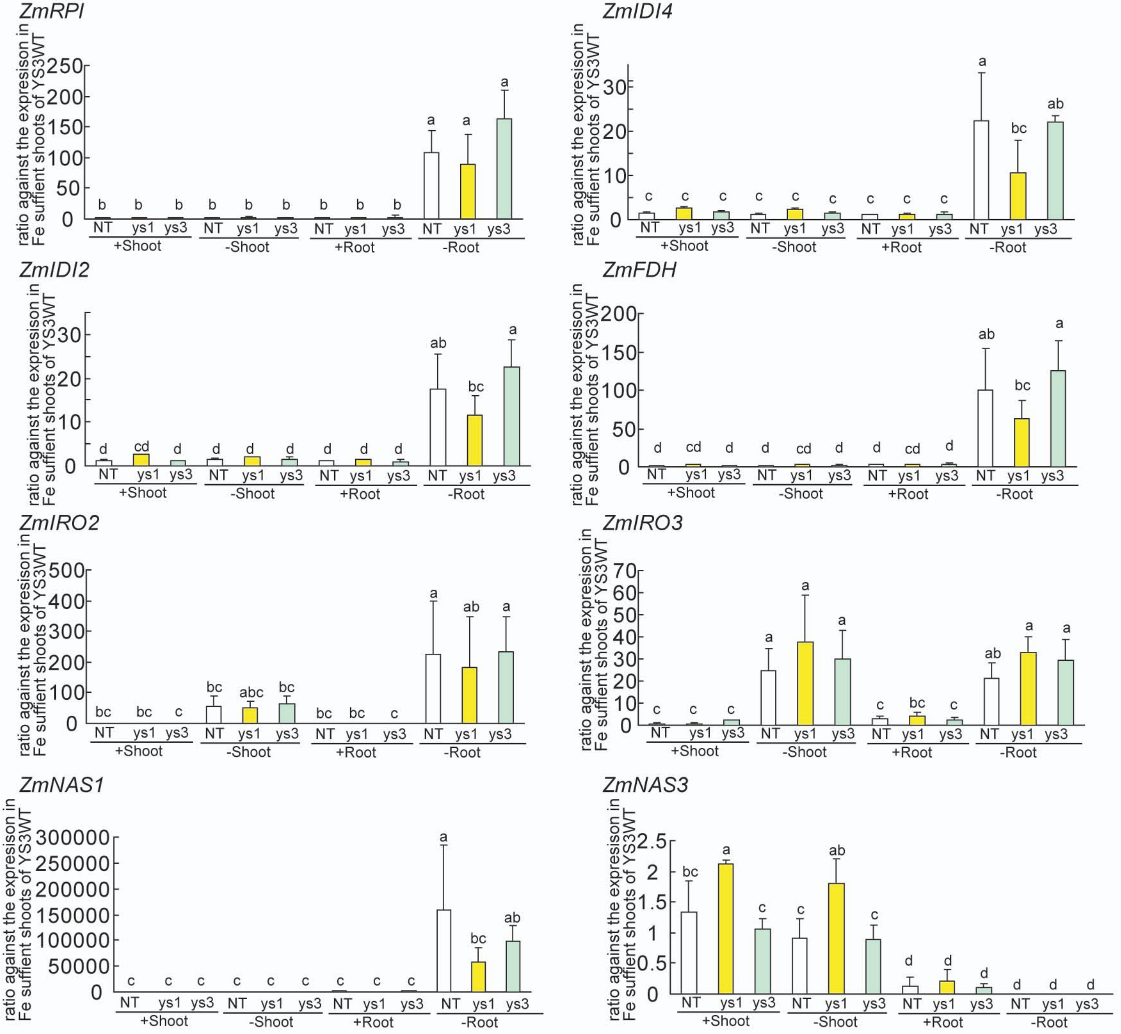

Figure S2. Biological replicates for quantitative real-time PCR for the the difference in the expression levels of the Fe-deficiency-inducible genes among the *YS1,YS3* [wild type (YS3WT), which is the same cultivar and has the same genetic background with ys3 mutant] and *ys1* or *ys3* mutant. The quantitative real-time PCR of the genes involved in the methionine cycle (*ZmIDI2*, GRMZM2G139533; *ZmFDH*, GRMZM2G049811; *ZmIDI4*, GRMZM2G067265; *ZmRPI*, GRMZM2G035599), transcription (*ZmIRO2*, GRMZM2G057413; *ZmIRO3*, GRMZM2G350312), and MAs biosynthesis (*ZmNAS1*, GRMZM2G034956; *ZmNAS3*, GRMZM2G478568) was performed with appropriate primers (Table1S in File S1). The data are shown as the ratio against the expression in Fe-sufficient YS3WT shoots. The ubiquitin gene (UBQ) was used to normalize data. S.D. was culculated from the biological replicates (n=3-6). Column bars followed by different letters are significantly different from each other according to the Turkey-Kramer HSD test (n=3-6, $P<0.05$).+, Fe-sufficient condition; –, Fe-deficient condition.

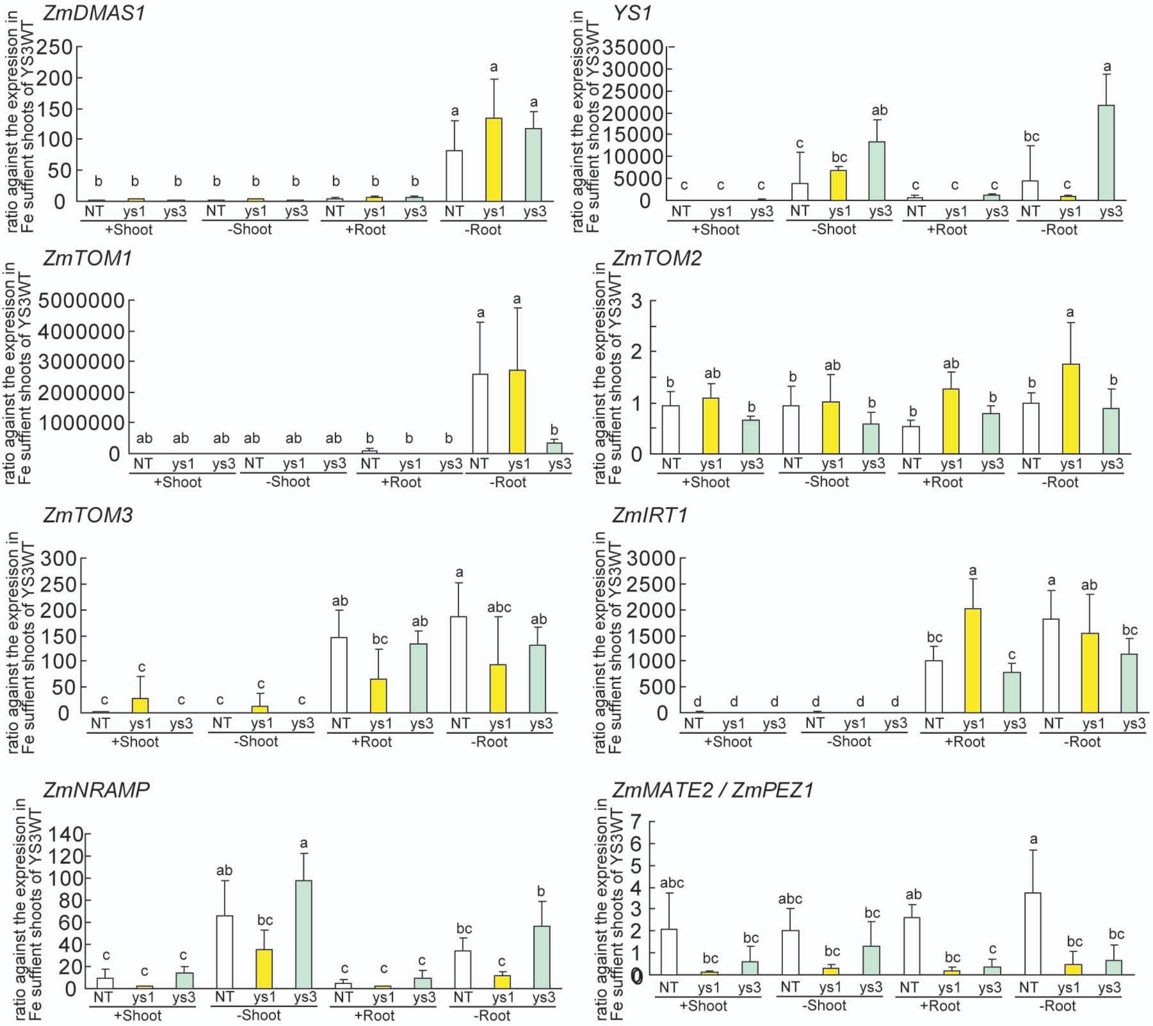

Figure S3. Biological replicates for quantitative real-time PCR for the the difference in the expression levels of the Fe-deficiency-inducible genes among the *YS1,YS3* [wild type (YS3WT), which is the same cultivar and has the same genetic background with *ys3* mutant] and *ys1* or *ys3* mutant. The quantitative real-time PCR of the genes involved in MAs biosynthesis (*ZmDMAS1*, GRMZM2G060952) and transport (*ZmYS1*, GRMZM2G156599; *ZmTOM1*, GRMAM2G063306; *ZmTOM2*, GRMZM5G877788; *ZmTOM3*, GRMZM2G141081; *ZmIRT1*, GRMZM2G118821; *ZmNRAMP1*, GRMZM2G178190; *ZmMATE2/ZmPEZ1*, GRMZM2G170128) was performed with appropriate primers (Table S1 in File S1). The data are shown as the ratio against the expression in Fe-sufficient YS3WT shoots. The ubiquitin gene (UBQ) was used to normalize data. S.D. was culculated from the biological replicates (n=3-6). Column bars followed by different letters are significantly different from each other according to the Turkey-Kramer HSD test (n=3-6, P<0.05).+, Fe-sufficient condition; –, Fe-deficient condition.

Figure S4. Unspliced introns in *ZmTOM1* of *ys3*. GRMZM2G063306_T02orf, *ZmTOM1* cDNA sequence predicted by GRAMENE; ZmTOM1pcr, partial sequence of *ZmTOM1* which was used for qRT-PCR; ZmTOM1orf, Sequenced *ZmTOM1* from WT; Pattern_1, intron insertion version of *ZmTOM1* cDNA in *ys3*; Pattern_2, intron insertion version of *ZmTOM1* cDNA in *ys3*; Pattern_3, intron insertion version of *ZmTOM1* cDNA in *ys3*.

```
//

                          1                                                   50
GRMZM2G063306_T02orf      .......... ATGGCTGAGG AGGTGCCAAC GACGCCGCCG CCGGCACCAT
ZmTOM1pcr                 .......... .......... .......... .......... ..........
ZmTOM1orf                 .......... ATGGCTGAGG AGGTGCCAAC NACGCCGCCG CCGGCACCAT
Pattern_1                 .......... .......... .......... .......... ..........
Pattern_2                 .......... .......... .......... .......... ..........
Pattern_3                 .......... .......... .......... .......... ..........

                          51                                                 100
GRMZM2G063306_T02orf      TGTACGTCGA TGGCTGTCCG GGATGCGCCA TGGACCGGAA GAAGGCGGCC
ZmTOM1pcr                 .......... .......... .......... .......... ..........
ZmTOM1orf                 TGTACGTCGA TGGCTGTCCG GGATGCGCCA TGGACCGGAA GAAGGCGGCC
Pattern_1                 .......... .......... .......... .......... ..........
Pattern_2                 .......... .......... .......... .......... ..........
Pattern_3                 .......... .......... .......... .......... ..........

                          101                                                150
GRMZM2G063306_T02orf      AACAAGGGCA TCCCCTACAA GGAGTTCTTC TTCGTCGCAG TCACCACCAT
ZmTOM1pcr                 .......... .......... .......... .......... ..........
ZmTOM1orf                 AACAAGGGCA TCCCCTACAA GGAGTTCTTC TTCGTCGCAG TCACCACCAT
Pattern_1                 .......... .......... .......... .......... ..........
Pattern_2                 .......... .......... .......... .......... ..........
Pattern_3                 .......... .......... .......... .......... ..........

                          151                                                200
GRMZM2G063306_T02orf      TGCCTCTGCT TTGCCAATAT CGTCCCTGTT TCCCTTCTTG TATTTCATGA
ZmTOM1pcr                 .......... .......... .......... .......... ..........
ZmTOM1orf                 CGCCTCTGCT TTGCCAATAT CGTCCCTGTT TCCCTTCTTG TATTTCATGA
Pattern_1                 .......... .......... .......... .......... ..........
Pattern_2                 .......... .......... .......... .......... ..........
Pattern_3                 .......... .......... .......... .......... ..........

                          201                                                250
GRMZM2G063306_T02orf      TCGAAGATCT ACACGTGGCC AAAAAAGAAC AAGATATTGG ACTGTACGCT
ZmTOM1pcr                 .......... .......... .......... .......... ..........
ZmTOM1orf                 TCGAAGATCT ACACGTGGCC AAAAAAGAAC AAGATATTGG ACTGTACGCT
```

```
Pattern_1              .......... .......... .......... .......... ..........
Pattern_2              .......... .......... .......... .......... ..........
Pattern_3              .......... .......... .......... .......... ..........

                       251                                                300
GRMZM2G063306_T02orf   GGTTTTCTTG GTGCATCTTA CTTTGTCGGT CGATTTTTGG TCTCGTTCTT
ZmTOM1pcr              ..TTTTCTTG GTGCATCTTA CTTTGTCGGT CGATTTTTGG TCTCGTTCTT
ZmTOM1orf              GGTTTTCTTG GTGCATCTTA CTTTGTCGGT CGATTTTTGG TCTCGTTCTT
Pattern_1              ..TTTTCTTG GTGCATCTTA CTTTGTCGGT CGATTTTTGG TCTCGTTCTT
Pattern_2              ..TTTTCTTG GTGCATCTTA CTTTGTCGGT CGATTTTTGG TCTCGTTCTT
Pattern_3              ..TTTTCTTG GTGCATCTTA CTTTGTCGGT CGATTTTTGG TCTCGTTCTT

                       301                                                350
GRMZM2G063306_T02orf   CTGGGGTGTG GTGGCTGATC GTGTCGGAAG GAAGCCCATC ATTATATTTT
ZmTOM1pcr              CTGGGGTGTG GTGGCTGATC GTGTCGGAAG GAAGCCCATC ATTATATTTT
ZmTOM1orf              CTGGGGTGTG GTGGCTGATC GTGTCGGAAG GAAGCCCATC ATTATATTTT
Pattern_1              CTGGGGTGTG GTGGCTGATC GTGTCGGAAG GAAGCCCATA ATTATATTTT
Pattern_2              CTGGGGTGTG GTGGCTGATC GTGTCGGAAG GAAGCCCATA ATTATATTTT
Pattern_3              CTGGGGTGTG GTGGCTGATC GTGTCGGAAG GAAGCCCATA ATTATATTTT

                       351                                                400
GRMZM2G063306_T02orf   CAGTCGCTTC AGTGGTTGTA TTCAACACAC TGTTTGGATT AAGTGTACAA
ZmTOM1pcr              CAGTCGCTTC AGTGGTTGTA TTCAACACAC TGTTTGGATT AAGTGTACAA
ZmTOM1orf              CAGTCGCTTC AGTGGTCGTA TTCAACACAC TGTTTGGATT AAGTGTACAA
Pattern_1              CAGTCGCTTC AGTGGTCGTA TTCAACACAC TGTTTGGATT AAGTGTACAA
Pattern_2              CAGTCGCTTC AGTGGTCGTA TTCAACACAC TGTTTGGATT AAGTGTACAA
Pattern_3              CAGTCGCTTC AGTGGTCGTA TTCAACACAC TGTTTGGATT AAGTGTACAA


                       401                                                450
GRMZM2G063306_T02orf   TATTGGATGG CCATCACCAC AAGATTGCTT CTAGGTGCTC TCAATGGCAT
ZmTOM1pcr              TATTGGATGG CCATCACCAC AAGATTGCTT CTAGGTGCTC TCAATGGCAT
ZmTOM1orf              TATTGGATGG CCATCACCAC AAGATTGCTT CTAGGTGCTC TCAATGGCAT
Pattern_1              TATTGGATGG CCATCACCAC AAGATTGCTT CTAGGTGCTC TCAATGGCAT
Pattern_2              TATTGGATGG CCATCACCAC AAGATTGCTT CTAGGTGCTC TCAATGGCAT
Pattern_3              TATTGGATGG CCATCACCAC AAGATTGCTT CTAGGTGCTC TCAATGGCAT

                       451                                                500
GRMZM2G063306_T02orf   GCTTGCACCA ATAAAGG... .......... .......... ..........
ZmTOM1pcr              GCTTGCACCA ATAAAGG... .......... .......... ..........
ZmTOM1orf              GCTTGCACCA ATAAAGG... .......... .......... ..........
Pattern_1              GCTTGCACCA ATAAAGG... .......... .......... ..........
Pattern_2              GCTTGCACCA ATAAAGG... .......... .......... ..........
Pattern_3              GCTTGCACCA ATAAAGGTAG GTACCTTGAT TAGATTAGGA TGCAAAAAGT

                       501                                                550
```

```
GRMZM2G063306_T02orf  .......... .......... .......... .......... ..........
ZmTOM1pcr             .......... .......... .......... .......... ..........
ZmTOM1orf             .......... .......... .......... .......... ..........
Pattern_1             .......... .......... .......... .......... ..........
Pattern_2             .......... .......... .......... .......... ..........
Pattern_3             TATGCCATGG CAGTTAGTTT CAAATTATAA ATATATATAA ACAGGTTTGT

                      551                                               600
GRMZM2G063306_T02orf  .......... .......... .......... ..CTTACTCA ATCGAGATTT
ZmTOM1pcr             .......... .......... .......... ..CTTACTCA ATCGAGATTT
ZmTOM1orf             .......... .......... .......... ..CTTACTCA ATCGAGATTT
Pattern_1             .......... .......... .......... ..CTTACTCA ATCGAGATTT
Pattern_2             .......... .......... .......... ..CTTACTCA ATCGAGATTT
Pattern_3             TTATGACTCA TCTCTTTTTT ATTTTTTGCA GGCTTACTCA ATCGAGATTT

                      601                                               650
GRMZM2G063306_T02orf  GTCGACCTGA GCACCATGCT CTTGGGTTAT CAGTTGACAC GGTCTCAAGA
ZmTOM1pcr             GTCGACCTGA GCACCATGCT CTTGGGTTAT CAGTTGACAC GGTCTCAAGA
ZmTOM1orf             GTCGACCTGA GCACCATGCT CTTGGGTTAT CAGTTGTAAG CACGGCTTGG
Pattern_1             GTCGACCTGA GCACCATGCT CTTGGGTTAT CAGTTGTAAG CACGGCTTGG
Pattern_2             GTCGACCTGA GCACCATGCT CTTGGGTTAT CAGTTGTAAG CACGGCTTGG
Pattern_3             GTCGACCTGA GCACCATGCT CTTGGGTTAT CAGTTGTAAG CACGGCTTGN

                      651                                               700
GRMZM2G063306_T02orf  GCCACAAACA TACTATACC. .......... .......... ..........
ZmTOM1pcr             GCCACAAACA TACTATACC. .......... .......... ..........
ZmTOM1orf             GGCTTAGGTC TTATTGTTGG CCCGTACATT GGAGGCTACT TGGCGCAGCC
Pattern_1             GGCCTAGGTC TTATTGTTGG CCCGTCCATT GGAGGCTACT TGGCGCAGCC
Pattern_2             GGCCTAGGTC TTATTGTTGG CCCGTCCATT GGAGGCTACT TGGCGCAGCC
Pattern_3             GGCCTAGGTC TTATTGTTGG CCCGTCCATT GGAGGCTACT TGGCGCAGCC

                      701                                               750
GRMZM2G063306_T02orf  .......... .......... .......... .......... ..........
ZmTOM1pcr             .......... .......... .......... .......... ..........
ZmTOM1orf             TGCACGGCAA TATCCAAACG TATTCTCTGA GGAATCGATT TTTGGGAGGT
Pattern_1             TGCACGGCAA TATCCAAACG TATTCTCTGA GGAATCGATT TTTGGNAGGT
Pattern_2             TGCACGGCAA TATCCAAACG TATTCTCTGA GGAATCGATT TTTGGGAGGT
Pattern_3             TGCACGGCAA TATCCAAACG TATTCTCTGA GGAAATCGAT TTTTGGGAGG

                      751                                               800
GRMZM2G063306_T02orf  .......... .......... .......... .......... ..........
ZmTOM1pcr             .......... .......... .......... .......... ..........
ZmTOM1orf             TCCCGTATCT GCTGCCATGC CTAAGTATCT CCATTTTTTC TGCTGTCGTT
Pattern_1             TCCCGTATCT GCTGCCATGC CTNAGTATCT CCATTTTTTC TGCTGTCGTT
Pattern_2             TCCCGTATCT GCTGCCATGC CTAAGTATCT CCATTTTTTC TGCTGTCGTT
Pattern_3             TTCCCGTATC TGCTGCCATG CCTAAGTATC TCCATTTTTT CTGCTGTCGT
```

```
                      801                                                850
GRMZM2G063306_T02orf  .......... .......... .......... .......... ..........
ZmTOM1pcr             .......... .......... .......... .......... ..........
ZmTOM1orf             CTAGTAAGCT GCATATGGGT TACCGGGAGA CACTCCCATA AACATAAGAA
Pattern_1             CTAGTNATTA AGCTGCATTT GGNTACCGNA GANNCCCCAT AAACNTANNA
Pattern_2             CTAGTAATTA AGCTGCATAT GGTTACCGGA GACACTCCAT AAACATAAGA
Pattern_3             TCTAGTAATT AAGCTGCATA TGGGTTACCG GAAGACACTT CATAAACATA

                      851                                                900
GRMZM2G063306_T02orf  .......... .......... .......... .......... ..........
ZmTOM1pcr             .......... .......... .......... .......... ..........
ZmTOM1orf             TATAGAAAAC GAAGTAGGAA TGTCCGGACA CTCAAGGACT CCACAAACAG
Pattern_1             TATAGAAAAC GAAGTAGGAA TGTCCGGACA CTCAAGGACT CCACAAACAG
Pattern_2             TATAGAAAAC GAAGTAGGAA TGTCCGGACA CTCAAGGACT CCACAAACAG
Pattern_3             TATAGAAAAC GAAGTAGGAA TGTCCGGACA CTCAAGGACT CCACAAACAG

                      901                                                950
GRMZM2G063306_T02orf  .......... .......... .......... .......... ..........
ZmTOM1pcr             .......... .......... .......... .......... ..........
ZmTOM1orf             AAGACGTAGA TGCAGGCAAG AGTCTATATA AGAACTGGCC ATTGATGTCT
Pattern_1             AAGACGTAGA TGCAGGCAAG AGTCTATATA AGAACTGGCC ATTGATGTCT
Pattern_2             AAGACGTAGA TGCAGGCAAG AGTCTATATA AGAACTGGCC ATTGATGTCT
Pattern_3             AAGACGTAGA TGCAGGCAAG AGTCTATATA AGAACTGGCC ATTGATGTCT

                      951                                               1000
GRMZM2G063306_T02orf  .......... .......... .......... .......... ..........
ZmTOM1pcr             .......... .......... .......... .......... ..........
ZmTOM1orf             TCCATCATTG CGTACTGTGT TTTCACGCTT TCATGACACT GCATACACCG
Pattern_1             TCCATCATTG CGTACTGTGT TTTCACGCTT TCATGACACT GCATACACCG
Pattern_2             TCCATCATTG CGTACTGTGT TTTCACGCTT TCATGACACT GCATACACCG
Pattern_3             TCCATCATTG CGTACTGTGT TTTCACGCTT TCATGACACT GCATACACCG

                      1001                                              1050
GRMZM2G063306_T02orf  .......... .......... .......... .......... ..........
ZmTOM1pcr             .......... .......... .......... .......... ..........
ZmTOM1orf             AGATACTTTC CTTGTGGACC GTAAGTGACA AAAAGTATGG GTGGGCTAGG
Pattern_1             AGATACTTTC CTTGTGGACC GTAAGTGACA AAAAGTATGG GTGGGCTAGG
Pattern_2             AGATACTTTC CTTGTGGACC GTAAGTGACA AAAAGTATGG GTGGGCTAGG
Pattern_3             AGATACTTTC CTTGTGGACC GTAAGTGACA AAAAGTATGG GTGGGCTAGG

                      1051                                              1100
GRMZM2G063306_T02orf  .......... .......... .......... .......... ....GTCTTG
ZmTOM1pcr             .......... .......... .......... .......... ....GTCTTG
ZmTOM1orf             CTTTTCTTTC TAAGGGGAGT TGGGTCAACT TCTTGCGATT GCAGGTCTTG
Pattern_1             CTTTTCTTTC TAAGGGGAGT TGGGTCAACT TCTTGCGATT GCAGGTCTTG
```

```
Pattern_2             CTTTTCTTTC TAAGGGGAGT TGGGTCAACT TCTTGCGATT GCAGGTCTTG
Pattern_3             CTTTTCTTTC TAAGGGGAGT TGGGTCAACT TCTTGCGATT GCAGGTCTTG

                      1101                                              1150
GRMZM2G063306_T02orf  GGCTTATTGT GTATCAAATG TTCATCTACA GGCCAGTCCA CAGATATCTC
ZmTOM1pcr             GGCTTATTGT GTATCAAATG TTCATCTACA GGCCAGTCCA CAGATATCTC
ZmTOM1orf             GGCTTATTGT GTATCAAATG TTCATCTACA GGCCAGTCCA CAGATATCTC
Pattern_1             GGCTTATTGT GTATCAAATG TTCATCTACA GGCCAGTCCA CAGATATCTC
Pattern_2             GGCTTATTGT GTATCAAATG TTCATCTACA GGCCCAGTCC ACAGATATCT
Pattern_3             GGCTTATTGT GTATCAAATG TTCATCTACA GGCCAGTCCA CAGATATCTC

                      1151                                              1200
GRMZM2G063306_T02orf  GGAAGTGTAA ATTCATGCCG TGCTGCATCT GCGCTATCCA TACCACTTAT
ZmTOM1pcr             GGAAGTGTAA ATTCATGCCG TGCTGCATCT GCGCTATCCA TACCACTTAT
ZmTOM1orf             GGAAGTGTAA ATTCATGCCG TGCTGCATCT GCGCTATCCA TACCACTTAT
Pattern_1             GGAAGTGTAA ATTCATGCCG TGCTGCATCT GCGCTATCCA TACCACTTAT
Pattern_2             GGAAGTGTAA ATTCATGCCG TGCTGCATCT GCGCTATCCA TACCACTTAT
Pattern_3             GGAAGTGTAA ATTCATGCCG TGCTGCATCT GCGCTATCCA TACCACTTAT

                      1201                                              1250
GRMZM2G063306_T02orf  CGCTGCTTTT CCCTTCATGA CACACCTGTC AGGATCAAAA CTTGGACTAG
ZmTOM1pcr             CGCTGCTTTT CCCTTCATGA CACACCTGTC AGGATCAAAA CTTGGACTAG
ZmTOM1orf             CGCTGCTTTT CCCTTCATGA CACACCTGTC AGGATCAAAA CTTGGACTAG
Pattern_1             CGCTGCTTTT CCCTTCATGA CACACCTGTC AGGATCAAAA CTTGGACTAG
Pattern_2             CGCTGCTTTT CCCTTCATGA CACACCTGTC AGGATCAAAA CTTGGACTAG
Pattern_3             CGCTGCTTTT CCCTTCATGA CACACCTGTC AGGATCAAAA CTTGGACTAG

                      1251                                              1300
GRMZM2G063306_T02orf  CTCTTTATTT TGCTACTATT TTGAAAGCTG CTCTTGGGAT AA........
ZmTOM1pcr             CTCTTTATTT TGCTACTATT TTGAAAGCTG CTCTTGGGAT AA........
ZmTOM1orf             CTCTTTATTT TGCTACTATT TTGAAAGCTG CTCTTGGGAT AA........
Pattern_1             CTCTTTATTT TGCTACTATT TTGAAAGCTG CTCTTGGGGT AAGTAGTTCC
Pattern_2             CTCTTTATTT TGCTACTATT TTGAAAGCTG CTCTTGGGAT AA........
Pattern_3             CTCTTTATTT TGCTACTATT TTGAAAGCTG CTCTTGGGAT AA........

                      1301                                              1350
GRMZM2G063306_T02orf  .......... .......... .......... .......... ..........
ZmTOM1pcr             .......... .......... .......... .......... ..........
ZmTOM1orf             .......... .......... .......... .......... ..........
Pattern_1             AACACCTACT GAGCACAAAC TAAATCATTA ATTAGTTGGT CGATCAAGAA
Pattern_2             .......... .......... .......... .......... ..........
Pattern_3             .......... .......... .......... .......... ..........

                      1351                                              1400
GRMZM2G063306_T02orf  .......... .......... .......... .......... ..........
ZmTOM1pcr             .......... .......... .......... .......... ..........
```

```
ZmTOM1orf             .......... .......... .......... .......... ..........
Pattern_1             TTGTACCGTT CGAACTCATT ATTATTTTAT GTGGTCATGG GAAAGAAAAC
Pattern_2             .......... .......... .......... .......... ..........
Pattern_3             .......... .......... .......... .......... ..........

                      1401                                              1450
GRMZM2G063306_T02orf  .......... .......... .......... .......... ..........
ZmTOM1pcr             .......... .......... .......... .......... ..........
ZmTOM1orf             .......... .......... .......... .......... ..........
Pattern_1             AAAGAATCTA TGCATTTATT GTATATATTT TAATCTTTAT AGTTACAGTA
Pattern_2             .......... .......... .......... .......... ..........
Pattern_3             .......... .......... .......... .......... ..........

                      1451                                              1500
GRMZM2G063306_T02orf  .......... .......... .......... .......... ..........
ZmTOM1pcr             .......... .......... .......... .......... ..........
ZmTOM1orf             .......... .......... .......... .......... ..........
Pattern_1             TCATCTTTCA ATTTGTTAAC GTCGTTACAC CATTGACGAA TGTGGCCAGA
Pattern_2             .......... .......... .......... .......... ..........
Pattern_3             .......... .......... .......... .......... ..........

                      1501                                              1550
GRMZM2G063306_T02orf  ...CTATCCT GACAGGCACT TCACTTCTGC AGAACTATGC TGTG......
ZmTOM1pcr             ...CTATCCT GACAGGCACT TCACTTCTGC AGAACTATGC TGTG......
ZmTOM1orf             ...CTATCCT GACAGGCACT TCACTTCTGC AGAACTATGC TGTG......
Pattern_1             TAACTATCCT GACAGGCACT TCACTTCTGC AGAACTATGC TGTGGCAAGT
Pattern_2             ...CTATCCT GACAGGCACT TCACTTCTGC AGAACTATGC TGTG......
Pattern_3             .......... .......... .......... .......... ..........

                      1551                                              1600
GRMZM2G063306_T02orf  .......... .......... .......... .......... ..........
ZmTOM1pcr             .......... .......... .......... .......... ..........
ZmTOM1orf             .......... .......... .......... .......... ..........
Pattern_1             ATTCACACAT ATAAATATAT ATTGATTTAC CTGAGTTTTA TGTTTCAAGT
Pattern_2             .......... .......... .......... .......... ..........
Pattern_3             .......... .......... .......... .......... ..........

                      1601                                              1650
GRMZM2G063306_T02orf  .......... .......... .......... .......... .........C
ZmTOM1pcr             .......... .......... .......... .......... .........C
ZmTOM1orf             .......... .......... .......... .......... .........C
Pattern_1             GCCTCTCTAC AAGTTTTAAT TAGCTAATTC AGTTGACCTT CGAAAACAGC
Pattern_2             .......... .......... .......... .......... .........C
Pattern_3             .......... .......... .......... .......... ..........

                      1651                                              1700
```

```
GRMZM2G063306_T02orf  CACAGCA TCAAAGAGGC GCCGCAAACG GCGTAGCGGC GACTGCAATGTCC
ZmTOM1pcr             CACAGCA TCAAAGAGGC GCCGCAAACG GCGTAGCGGC GACTGCAATGTCC
ZmTOM1orf             CACAGCA TCAAAGAGGC GCCGCAAACG GCGTAGCGGC GACTGCAATGTCC
Pattern_1             CACAGCA TCAAAGAGGC GCCGCAAACG GCGTAGCGGC GACTGCAATGTCC
Pattern_2             CACAGCA TCAAAGAGGC GCCGCAAACG GCGTAGCGGC GACTGCAATGTCC
Pattern_3             CACAGCA TCAAAGAGGC GCCGCAAACG GCGTAGCGGC GACTGCAATGTCC

                      1701                                             1750
GRMZM2G063306_T02orf  TTCTTTAAGG CTATTGGACC TGCAGGAGCA GGCGCTTT.. ..........
ZmTOM1pcr             TTCTTTAAGG CTATTGGACC TGCAGGAGCA GGCGCTTT.. ..........
ZmTOM1orf             TTCTTTAAGG CTATTGGACC TGCAGGAGCA GGCGCTTT.. ..........
Pattern_1             TTCTTTAAGG CTATTGGACC TGCAGGAGCA GGCGCTTT.. ..........
Pattern_2             TTCTTTAAGG CTATTGGACC TGCAGGAGCA GGCGCTTTGT GAGTATTGTC
Pattern_3             TTCTTTAAGG CTATTGGACC TGCAGGAGCA GGCGCTTT.. ..........

                      1751                                             1800
GRMZM2G063306_T02orf  .......... .......... .......... .......... ..........
ZmTOM1pcr             .......... .......... .......... .......... ..........
ZmTOM1orf             .......... .......... .......... .......... ..........
Pattern_1            .......... .......... .......... .......... ..........
Pattern_2             CCCCACGCAT CATATATGCT TTATTCGAAT ACAGATTATA TATAATATAT
Pattern_3             .......... .......... .......... .......... ..........

                      1801                                             1850
GRMZM2G063306_T02orf  .......... .......... .......... .......... ..........
ZmTOM1pcr             .......... .......... .......... .......... ..........
ZmTOM1orf             .......... .......... .......... .......... ..........
Pattern_1             .......... .......... .......... .......... ..........
Pattern_2             GAATTGTTGA ATATGATATG TCAAACATCT TCTCCTCCAA GAGGCAGATC
Pattern_3             .......... .......... .......... .......... ..........

                      1851                                             1900
GRMZM2G063306_T02orf  .......... .......... .......... .......... ..........
ZmTOM1pcr             .......... .......... .......... .......... ..........
ZmTOM1orf             .......... .......... .......... .......... ..........
Pattern_1             .......... .......... .......... .......... ..........
Pattern_2             GAATTGATCG ATGCACCGTG CACGTCATAT TTAAGTGTGT TAGTACCTTG
Pattern_3             .......... .......... .......... .......... ..........

                      1901                                             1950
GRMZM2G063306_T02orf  .......... .......... .......... .......... ..........
ZmTOM1pcr             .......... .......... .......... .......... ..........
ZmTOM1orf             .......... .......... .......... .......... ..........
Pattern_1             .......... .......... .......... .......... ..........
Pattern_2             TTTGTCTAGT ATCACAAATC CATGCTAGTT CCAGATTCGT GTGCACCATT
Pattern_3             .......... .......... .......... .......... ..........
```

```
                       1951                                              2000
GRMZM2G063306_T02orf   .......... .......... .......... .......... ..........
ZmTOM1pcr              .......... .......... .......... .......... ..........
ZmTOM1orf              .......... .......... .......... .......... ..........
Pattern_1              .......... .......... .......... .......... ..........
Pattern_2              ATTCATGCAT TAAACTCATC AAAAATCTGA TTGATGAGGC TTTTTAATGT
Pattern_3              .......... .......... .......... .......... ..........

                       2001                                              2050
GRMZM2G063306_T02orf   ..ATTCTCAT GGACCCAAAA ACGCCAAGAT GCTGCCTTCT TTCCAGGGGA
ZmTOM1pcr              ..ATTCTCAT GGACCCAAAA ACGCCAAGAT GCTGCCTTCT TTCCAGGGGA
ZmTOM1orf              ..ATTCTCAT GGACCCAAAA ACGCCAAGAT GCTGCCTTCT TTCCAGGGGA
Pattern_1              ..ATTCTCAT GGACCCAAAA ACGCCAAGAT GCTGCCTTCT TTCCAGGGGA
Pattern_2              AGATTCTCAT GGACCCAAAA ACGCCAAGAT GCTGCCTTCT TTCCGAGGGG
Pattern_3              .......... .......... .......... .......... ..........

                       2051                                              2100
GRMZM2G063306_T02orf   TCACATGTTG TTCTTCATTC TGAATTTGGT CCAATGTATT GGGCTCGTGC
ZmTOM1pcr              TC........ .......... .......... .......... ..........
ZmTOM1orf              TCACATGTTG TTCTTCATTC TGAATTTGGT CCAATTTATT GGGCTCGCGC
Pattern_1              TC........ .......... .......... .......... ..........
Pattern_2              TC........ .......... .......... .......... ..........
Pattern_3              .......... .......... .......... .......... ..........

                       2101                                              2150
GRMZM2G063306_T02orf   TGACCCTTAA ACCATTCCTA GCAGTACCGA AACACTATAA A.........
ZmTOM1pcr              .......... .......... .......... .......... ..........
ZmTOM1orf              TGACCCTTAA ACCATTCCTA GCAGTACCGA AACACTATAA A.........
Pattern_1              .......... .......... .......... .......... ..........
Pattern_2              .......... .......... .......... .......... ..........
Pattern_3              .......... .......... .......... .......... ..........
```

Figure S5. Genomic sequence of GRMZM2G063306. The colored and bold font is ensemble plants exons in this region. The pink color fonts show the region where sequencing was not perfect.

>chromosome:AGPv2:3:112041504:112048082:-1
CCAGGGTTTGACTCCGCATATATAAATAGCTTCCTCTCCGTCAGCGCTGACACAGTACGT
CTTACTAACCATATTATGATACTCAAATTACCTGCCCACCATCCCCTGCAAAACTAGCCA
CACATATATGTAAGTATGTGTATTTGTTTGCTGCAGCGCTTCTCCTTCAAGTACGTACGT
GTCGCCTGTGCCAATGGATAATTTGATTTGCCTCGGCATAAATATATACAAACTGTGTGG
CCTAGCTAGCTTACCAACCGTCTTTTGTCCTTGTATACTATGACAACAAGTTGGTTATAC
TCCGCGCGCCTTGCATCAGGAAATCGACGTAGTGCAAAAGCGGGCGGGAGGGCGGCGGCA
TGTGCATGCATGCCGCCCATCGGCGCGTACGCATATGCCACGCACGCTACACAGAGACGT
GCGTACGTACGTAGAGCAGTCTCGCTCGTCCAGCTCGATCTACACAACATCTGGCTGACT
AGCTACATGCAGTGTGCATGTCGTCATTATTACTCACCTCGGTAGTAGCATTACAATGTA
TGTATGTATATATAACCCATGCAGGCAGCTACCACCCGGCCAAGAGCAGTATTCCAGGCC
CAGGGTGTGTGTGTGCTGCTAAACCACCAAGGTCGAAGTATCCCTTACTGCTTGGTATAC
GTACGAGTAGCGTGTCCCTGTCCATTGTACGTACGTCGATGGCTGAGGAGGTGCCAACGA
CGCCGCCGCCGGCACCATTGTACGTCGATGGCTGTCCGGGATGCGCCATGGACCGGAAGA
AGGCGGCCAACAAGGGCATCCCCTACAAGGAGTTCTTCTTCGTCGCAGTCACCACCATTG
CCTCTGGTACCATATGGGACGATCGGATATATATGCTCTTTATCTGTTGCTGGCCTTTCC
TCTTCTGAAGATCATAATTGCCGCCCTCTCGATGCATCACTCTCTGTTCCTTTCACATGT
ACATTATGCATGCTTTTCTATGCCATCCCATGCTTTACATGCCGCATCGTCGACGTCATC
TCCCCTCGCACAAAATAATTAGAGACTCTCTTCTTGATGATGATCCAATATATTTGAAGA
CACAAGTGTCTTTTTTTTTGTTCCATCAAAACACCTATTCATATGCATGGTACGTAGCCCT
TCATCTCCTTCATGTATTGTGGCGAGATACGGACTAAATGTTGGTTGTGGAACACACTTC
AGTTGCACTCTTGATAGCTATATGACTCAGCATTTTACTTAAAACATGTACTATGTGTAT
ATCAAGAGTTATTGTGTTGCTCGGTAATTCATCATGTGTTTCTTTTTTTCTAAAACTAGTG
TGTGGGGATATAATAGAAGCTAGTTGATATATCAACTTGAACTATATCATACTTGCCCAT
GAATTGAATTCATCCTCTAGCATGGTAGTATGTATCTTAAGTATTAAAGTTTCATTGATA
AATCTACTATATGCACTAGTTCGAAATTGATTATTTTCAGTCCTCAACCTAATGCACATT
TATATATATTATGTAATTTCTATTCGGTTGTGATAAATCCACCAATTACACTCCATATGT
GTTGTCATCATTTGCATTGAACTTTTAAGGGACACGCAATATGTATTAAACCAAAGTTAT
ATCAACTCATGCAGCATTATTATTCAATATTTGGTTTTTAATTTTTGATCTTACAAGTTG
TTGCAATCTTTTTATCCATGGGCTAAGTTCAAACTTTAGTTTATTCAAAATTTTTCCCTT
TTATTTTAGTAAACACATGCTATAAGTTCTAGATTACATTATTTTTTTGGTGAATCATAGT
ACAACAAAGACATACATGTCCATACATTCACCCCTTCACCTTACCTCTACCCACATCACC
CGAGATTGTTAAAGTCATCACATGTGCATCGTTGTCATCGAAAACATCGCCTACCACAAA
AAGGACAACATTAAATCCAAGAATAAATTCAAGAAAACAAAATGCGAGCATCCGTCTCAA
GTCGAGGAGTTTAAGCTCGAGCGATCAGGTTCCACCATGAGGAATCCAACAAGCTACTAT
GCTCAGTTTGCTTCTAGATTCCATACTCAGTTGCTATAGTAATATTGTTTTATATAACGT
AGACTATTTAAAATAAATTTTGTCATAATTTTCCCTAAATTTACCGAAATAAAAAATTTA
TAAAGTTGCTTCTCATGAGTATTAATATAATATAATTTATTATATCAATGTCATGTGACT
CGTTAAGGATACACTTTTCCACATTGACAAACTTATAAATCAATATGGTAGTTATTATTG
GTGGTTAGAGCAATTAAATTAAAGGCTATATGTTTCTGATTTTTTTTCTTCAGAATTTCT
ATTATTATCTCGTTTTTCAATCAATTGAGGGTCTCCAATTATATTATTATTAAGATGGCA
GGATTAGTATTTTCTGTAGATTCTACAAACAATATCCATGATAGCATGAAGATCTCACTA
TTGTAATAATGCTGCTTACATGCACCATGCATGACTAATCATGCATTTATATGTGCTAGT

TATTTAGAAAATATTATATTTACCCATATATACAAGTGACTTGTTTCCTTATACATTGAC
AATATGTTCCTTTTCAG**CTTTGCCAATATCGTCCCTGTTTCCCTTCTTGTATTTCATG**GT
AGGTCGGTTATTATTGACCTCACTTTAACTTTTAAAAAAACATTTTTTTTTTTATTATGA
GTAGTAAACATAGTTCTTCATGACACAATAACATATTTATAG**ATCGAAGATCTACACGTG**
GCCAAAAAAGAACAAGATATTGGACTGTACGCTGGTTTTCTTGGTAAGAGGGTCACATCG
TCGTAATATATGCTAACAAACATACGGCAATGGCTTATGTTTGTACTGCATATATATACA
G**GTGCATCTTACTTTGTCGGTCGATTTTTGGTCTCGTTCTTCTGGGGTGTGGTGGCTGAT**
CGTGTCGGAAGGAAGCCCATCATTATATTTTCAGTCGCTTCAGTGTGAGTACAATTAATT
TGAAACGGATCATTAATTATGGCTGCAGCTTTCTTAATATAAACTCTGTAACACAAAAAA
ATGGTACGTTTTTCGATTTCAG**GGTTGTATTCAACACACTGTTTGGATTAAGTGTACAAT**
ATTGGATGGCCATCACCACAAGATTGCTTCTAGGTGCTCTCAATGGCATGCTTGCACCAA
TAAAGGTAGTAGGTACCTTGATTAAATTAGGATGCAAAAATTTATGCCATGGCAGTTAGT
TTCAAATTATAAATATATATAAACAGGTTTGTTTATGACTCATCTCTTCTTCTTTTTTTT
TTTTTGCAG**GCTTACTCAATCGAGATTTGTCGACCTGAGCACCATGCTCTTGGGTTATCA**
GTTGTAAGTAAATATCAACCCGTACTTTCTAATATATTGGAAGCATGCAGCTATTAATTG
CTCGAGTTGAGATTAATAACGTACTGAATACAG**GTAAGCACGGCTTGGGGCCTAGGTCTT**
ATTGTTGGCCCGTCCATTGGAGNNNNNNNNNNNNNNNNNNNNNNNNNNNNNNNNNNNNNN
NNNNNNNNNNNNNNNNNNNNNNNNNNNNNNNNNNNNNNNNNNNNNNNNNNNNNNNNNNNN
NNCGAGGTAATTATTTTTAATAGTTTATATATATATATATATATATATATATATATATAT
ATATATATATATATATATATACCTGAATCTATCATTTGAATCAATTGAACAAAAATGGGA
GCATCTTTGTTAAAACAGATACTTTCCTTGTGGACCGTAAGTGACAAAAAGTATGGTGGG
CTAGGCTTTTCTTCTAAGGGAGTTGGTCAACTTCTTGCGATTGCAGGTATGTTCATTTGT
AAGGTGTAAATAATTCTATAATACGTACAATAGCTGCTACCAGACGTTGTTTACTATTAA
TTTTTTTCCGTTTAATATTTTTCAGATACATAGTGGATTTTGCTATTTATCTAGATATGT
AGCTTCTACTACATATCTATACAAGACAAAACATATTTACAATTTGGAATGGATAAAATG
TTATTTTTCCTCAACCCAGGATATTAAGAGCATCTGCAACAATGCTTTAAACTGATGGCT
CAAATTGAAATATATGGCTCTACGCAGAAAAACTACTCAAAAAATGTCTAATTTTATAAA
ATTTGATTAAAAATTATAG**GACACGGTCTCAAGAGCCACAAACATACTATACC**GTACGTA
GTGAGTTGCCCTATAATATAGATTTGAGGATTTTAGAAATAAAATATTTTGTTGGTGCCT
TAAATTGTATAAAATATATACCCATTTTTAAATTATTGGTCATTTTTATAAATCATGTTG
TTACAGATGCTCTAATTTGACCTTCTCTTTCTCTGTGTTGTAG**GTCTTGGGCTTATTGTG**
TATCAAATGTTCATCTACAGGCCAGTCCACAGATATCTCGGAAGTGTAAATTCATGCCGT
GCTGCATCTGTGGGTGCCTAATAATAACCTGAAAAAAAATAGAAACGTGTGCATCACTCC
TCTATATCCACTAACTATTGTCATGTAAACTAAACTGCAG**GCGCTATCCATACCACTTAT**
CGCTGCTTTTCCCTTCATGACACACCTGTCAGGATCAAAACTTGGACTAGCTCTTTATTT
TGCTACTATTTTGAAAGCTGCTCTTGGGGTATGTAGTTCCAACACCTACTGAGCACAAAC
TAAATCATTAATTAGTTGGTCGATCAAGAATTGTACCGTTCCAACTCATTATTATATATT
ATTAATCTTTATAGTTACAGTATCATCTTTCAATTTGTTAACGTCGTTACACCTTTGACG
AATGTGGCCAG**ATAACTATCCTGACAGGCACTTCACTTCTGCAGAACTATGCTGTG**GCAA
GTATTCACACATATAAATATATATTGATTTACCTGAGTTTTATGTTTCAAGTGCCTCTCT
GCAAGTTTTAATTAGCTAATTCAGTTGACCTTTGAAAACAG**CCACAGCATCAAAGAGGCG**
CCGCAAACGGCGTAGCGGCGACTGCAATGTCCTTCTTTAAGGCTATTGGACCTGCAGGAG
CAGGCGCTTTGTGAGTATTGTCCCCCACGCATCATATATGCGTCCCGATTTTATTCGAAT
ACAGATTATATATAATATATGAATTGTTGAATATGATATGTCAAACATCTTCTCCTCCAA
GAGGCAGATCGAATTGATCGATGCACGTCATATTTAAGTGTGTTAGTACCTTGTTTGTCT
AGTATCACAAATCCATGCTAGTTCCAGATTCGTGTGCACCATTATTCATGCATTAAACTC

ATCAAAAATCTGATTGATGAGGCTTTTTAATGTAG**ATTCTCATGGACCCAAAAACGCCAA**
GATGCTGCCTTCTTTCCAGGTATATTTTTAAGTTACTTTTCTCTACTATATATAAATCAT
CAGTTCCCACGGTTCCGTTTTGACTGTCGTCATGCTTTCTTTCTCCTCATTTAAATAGTA
AAATAAAATTAAACCTATATTCACATCTATCTAGCCAGCAAAACACATAAGTCTTTATGA
TTTATGTTCTGTACTTAAGCATAAATAAAAACTGCAGTAACTTTGGTCGTTGTCTTTAAT
TTATGCACCACCCTAGTACCTGTTTTTTCTTTAATGTATTTATCTTATGTGTAGCACCAA
CTATGTTTTTATATTTGTGTTCCGATTGCCAAGAGAAAAATTAATTAATTCATTAATGAC
GCAG**GGGATCACATGTTGTTCTTCATTCTGAATTTGGTCCAATGTATTGGGCTCGTGCTG**
ACCCTTAAACCATTCCTAGCAGTACCGAAACACTATAAATGACAGCTGCTCGAACCCAAG
CAATGGAATAATGATGGATTAGCAAAACATATGTGCCCAAAAATACGTTTTTGAGTGGCA
TACAACGGAATTATGTAAAAGCTGTCAAGAAAAAGTAGCTACAATGATTGTGTCCGCTAT
CTTTACATCTCCTTACATTGAAGTTTTAATTTGAGCACATTTTGTGTCCAGTACATGGTA
TCCGCTGTTTCAATTGAAATGCTGGTTTGAGTATGAATGATGAAAAATCGAAGTTTAGAG
TTTAGTCATACAACATGTCAAAGTCAAGTGGCAGTACTATACCCTTGAAAGAGAACCCAA
GCGCATGCAACATCAGCGCCTGGCCCATCACTATCCATTGCTGATTTGTGGTTTGAATAG
ACAATGATAAGTGTTGGTGGTTCTTAGCCTAACAAACTTTACTCCGCAAGCGTACGGAGA
CTGTTGTAGCTTTCACCCCGAAATATTCTTGTGTATCATATTTATCCACAGGTAAGCATT
GACAAAGAATGATGATTCATCAATCATCTAGCTTCTACTAACTTATTCATCCCAAACGAT
AGGTAAAAGGTAAACACCAAACACCATGAGATACAAGCCAAGCACCGTTGGCATGAAAAC
ATGTTTAACTAGTAGGACAACGAGTCAAGACTAAGGTTTTGGTCTTCATTCTCAACCACA
TACAAGAGCTCTTCGTGCAAAAATAATCTTAACAGAGCTATGATCTTACGTCGAGCAAAA
TTTTGAGGCCCCAACGTGGTCGGGGGTAAATAGTGGCCA

Figure S6. Full length cDNA of *ZmTOM1*. GRMZM2G063306_T02orf, *ZmTOM1* cDNA sequence predicted by GRAMENE; ZmTOM1pcr, partial sequence of *ZmTOM1* which was used for qRT-PCR; ZmTOM1orf, Sequenced *ZmTOM1* from WT.

```
//

                         1                                                 50
GRMZM2G063306_T02orf     .......... ATGGCTGAGG AGGTGCCAAC GACGCCGCCG CCGGCACCAT
ZmTOM1pcr                .......... .......... .......... .......... ..........
ZmTOM1orf                .......... ATGGCTGAGG AGGTGCCAAC NACGCCGCCG CCGGCACCAT

                         51                                               100
GRMZM2G063306_T02orf     TGTACGTCGA TGGCTGTCCG GGATGCGCCA TGGACCGGAA GAAGGCGGCC
ZmTOM1pcr                .......... .......... .......... .......... ..........
ZmTOM1orf                TGTACGTCGA TGGCTGTCCG GGATGCGCCA TGGACCGGAA GAAGGCGGCC

                         101                                              150
GRMZM2G063306_T02orf     AACAAGGGCA TCCCCTACAA GGAGTTCTTC TTCGTCGCAG TCACCACCAT
ZmTOM1pcr                .......... .......... .......... .......... ..........
ZmTOM1orf                AACAAGGGCA TCCCCTACAA GGAGTTCTTC TTCGTCGCAG TCACCACCAT

                         151                                              200
GRMZM2G063306_T02orf     TGCCTCTGCT TTGCCAATAT CGTCCCTGTT TCCCTTCTTG TATTTCATGA
ZmTOM1pcr                .......... .......... .......... .......... ..........
ZmTOM1orf                CGCCTCTGCT TTGCCAATAT CGTCCCTGTT TCCCTTCTTG TATTTCATGA

                         201                                              250
GRMZM2G063306_T02orf     TCGAAGATCT ACACGTGGCC AAAAAAGAAC AAGATATTG GACTGTACGCT
ZmTOM1pcr                .......... .......... .......... .......... ..........
ZmTOM1orf                TCGAAGATCT ACACGTGGCC AAAAAAGAAC AAGATATTG GACTGTACGCT

                         251                                              300
GRMZM2G063306_T02orf     GGTTTTCTTG GTGCATCTTA CTTTGTCGGT CGATTTTTGG TCTCGTTCTT
ZmTOM1pcr                ..TTTTCTTG GTGCATCTTA CTTTGTCGGT CGATTTTTGG TCTCGTTCTT
ZmTOM1orf                GGTTTTCTTG GTGCATCTTA CTTTGTCGGT CGATTTTTGG TCTCGTTCTT

                         301                                              350
GRMZM2G063306_T02orf     CTGGGGTGTG GTGGCTGATC GTGTCGGAAG GAAGCCCATC ATTATATTTT
```

```
ZmTOM1pcr             CTGGGGTGTG GTGGCTGATC GTGTCGGAAG GAAGCCCATC ATTATATTTT
ZmTOM1orf             CTGGGGTGTG GTGGCTGATC GTGTCGGAAG GAAGCCCATC ATTATATTTT

                      351                                              400
GRMZM2G063306_T02orf  CAGTCGCTTC AGTGGTTGTA TTCAACACAC TGTTTGGATT AAGTGTACAA
ZmTOM1pcr             CAGTCGCTTC AGTGGTTGTA TTCAACACAC TGTTTGGATT AAGTGTACAA
ZmTOM1orf             CAGTCGCTTC AGTGGTCGTA TTCAACACAC TGTTTGGATT AAGTGTACAA

                      401                                              450
GRMZM2G063306_T02orf  TATTGGATGG CCATCACCAC AAGATTGCTT CTAGGTGCTC TCAATGGCAT
ZmTOM1pcr             TATTGGATGG CCATCACCAC AAGATTGCTT CTAGGTGCTC TCAATGGCAT
ZmTOM1orf             TATTGGATGG CCATCACCAC AAGATTGCTT CTAGGTGCTC TCAATGGCAT

                      451                                              500
GRMZM2G063306_T02orf  GCTTGCACCA ATAAAGGCTT ACTCAATCGA GATTTGTCGA CCTGAGCACC
ZmTOM1pcr             GCTTGCACCA ATAAAGGCTT ACTCAATCGA GATTTGTCGA CCTGAGCACC
ZmTOM1orf             GCTTGCACCA ATAAAGGCTT ACTCAATCGA GATTTGTCGA CCTGAGCACC

                      501                                              550
GRMZM2G063306_T02orf  ATGCTCTTGG GTTATCAGTT GACACGGTCT CAAGAGCCAC AAACATACTA
ZmTOM1pcr             ATGCTCTTGG GTTATCAGTT GACACGGTCT CAAGAGCCAC AAACATACTA
ZmTOM1orf             ATGCTCTTGG GTTATCAGTT GTAAGCACGG CTTGGGGCTT AGGTCTTATT

                      551                                              600
GRMZM2G063306_T02orf  TACC...... .......... .......... .......... ..........
ZmTOM1pcr             TACC...... .......... .......... .......... ..........
ZmTOM1orf             GTTGGCCCGT ACATTGGAGG CTACTTGGCG CAGCCTGCAC GGCAATATCC

                      601                                              650
GRMZM2G063306_T02orf  .......... .......... .......... .......... ..........
ZmTOM1pcr             .......... .......... .......... .......... ..........
ZmTOM1orf             AAACGTATTC TCTGAGGAAT CGATTTTTGG GAGGTTCCCG TATCTGCTGC

                      651                                              700
GRMZM2G063306_T02orf  .......... .......... .......... .......... ..........
ZmTOM1pcr             .......... .......... .......... .......... ..........
ZmTOM1orf             CATGCCTAAG TATCTCCATT TTTTCTGCTG TCGTTCTAGT AAGCTGCATA

                      701                                              750
GRMZM2G063306_T02orf  .......... .......... .......... .......... ..........
ZmTOM1pcr             .......... .......... .......... .......... ..........
```

```
ZmTOM1orf             TGGGTTACCG GGAGACACTC CCATAAACAT AAGAATATAG AAAACGAAGT

                      751                                                800
GRMZM2G063306_T02orf  .......... .......... .......... .......... ..........
ZmTOM1pcr             .......... .......... .......... .......... ..........
ZmTOM1orf             AGGAATGTCC GGACACTCAA GGACTCCACA AACAGAAGAC GTAGATGCAG

                      801                                                850
GRMZM2G063306_T02orf  .......... .......... .......... .......... ..........
ZmTOM1pcr             .......... .......... .......... .......... ..........
ZmTOM1orf             GCAAGAGTCT ATATAAGAAC TGGCCATTGA TGTCTTCCAT CATTGCGTAC

                      851                                                900
GRMZM2G063306_T02orf  .......... .......... .......... .......... ..........
ZmTOM1pcr             .......... .......... .......... .......... ..........
ZmTOM1orf             TGTGTTTTCA CG CTTTCAT GACACTGCAT ACACCGAGAT ACTTTCCTTG

                      901                                                950
GRMZM2G063306_T02orf  .......... .......... .......... .......... ..........
ZmTOM1pcr             .......... .......... .......... .......... ..........
ZmTOM1orf             TGGACCGTAA GTGACAAAAA GTATGGGTGG GCTAGGCTTT TC TTTCTAA

                      951                                               1000
GRMZM2G063306_T02orf  .......... .......... .......... ..GTCTTGGG CTTATTGTGT
ZmTOM1pcr             .......... .......... .......... ..GTCTTGGG CTTATTGTGT
ZmTOM1orf             GGGGAGTTGG GTC AACTTC TTGCGATTGC AGGTCTTGGGGCTTATTGTGT

                      1001                                              1050
GRMZM2G063306_T02orf  ATCAAATGTT CATCTACAGG CCAGTCCACA GATATCTCGG AAGTGTAAAT
ZmTOM1pcr             ATCAAATGTT CATCTACAGG CCAGTCCACA GATATCTCGG AAGTGTAAAT
ZmTOM1orf             ATCAAATGTT CATCTACAGG CCAGTCCACA GATATCTCGG AAGTGTAAAT

                      1051                                              1100
GRMZM2G063306_T02orf  TCATGCCGTG CTGCATCTGC GCTATCCATA CCACTTATCG CTGCTTTTCC
ZmTOM1pcr             TCATGCCGTG CTGCATCTGC GCTATCCATA CCACTTATCG CTGCTTTTCC
ZmTOM1orf             TCATGCCGTG CTGCATCTGC GCTATCCATA CCACTTATCG CTGCTTTTCC

                      1101                                              1150
```

```
GRMZM2G063306_T02orf  CTTCATGACA CACCTGTCAG GATCAAAACT TGGACTAGCT CTTTATTTTG
ZmTOM1pcr             CTTCATGACA CACCTGTCAG GATCAAAACT TGGACTAGCT CTTTATTTTG
ZmTOM1orf             CTTCATGACA CACCTGTCAG GATCAAAACT TGGACTAGCT CTTTATTTTG

                      1151                                              1200
GRMZM2G063306_T02orf  CTACTATTTT GAAAGCTGCT CTTGGGATAA CTATCCTGAC AGGCACTTCA
ZmTOM1pcr             CTACTATTTT GAAAGCTGCT CTTGGGATAA CTATCCTGAC AGGCACTTCA
ZmTOM1orf             CTACTATTTT GAAAGCTGCT CTTGGGATAA CTATCCTGAC AGGCACTTCA

                      1201                                              1250
GRMZM2G063306_T02orf  CTTCTGCAGA ACTATGCTGT GCCACAGCAT CAAAGAGGCG CCGCAAACGG
ZmTOM1pcr             CTTCTGCAGA ACTATGCTGT GCCACAGCAT CAAAGAGGCG CCGCAAACGG
ZmTOM1orf             CTTCTGCAGA ACTATGCTGT GCCACAGCAT CAAAGAGGCG CCGCAAACGG

                      1251                                              1300
GRMZM2G063306_T02orf  CGTAGCGGCG ACTGCAATGT CCTTCTTTAA GGCTATTGGA CCTGCAGGAG
ZmTOM1pcr             CGTAGCGGCG ACTGCAATGT CCTTCTTTAA GGCTATTGGA CCTGCAGGAG
ZmTOM1orf             CGTAGCGGCG ACTGCAATGT CCTTCTTTAA GGCTATTGGA CCTGCAGGAG

                      1301                                              1350
GRMZM2G063306_T02orf  CAGGCGCTTT ATTCTCATGG ACCCAAAAAC GCCAAGATGC TGCCTTCTTT
ZmTOM1pcr             CAGGCGCTTT ATTCTCATGG ACCCAAAAAC GCCAAGATGC TGCCTTCTTT
ZmTOM1orf             CAGGCGCTTT ATTCTCATGG ACCCAAAAAC GCCAAGATGC TGCCTTCTTT

                      1351                                              1400
GRMZM2G063306_T02orf  CCAGGGGATC ACATGTTGTT CTTCATTCTG AATTTGGTCC AATGTATTGG
ZmTOM1pcr             CCAGGGGATC
ZmTOM1orf             CCAGAGGATC ACATGTTGTT CTTCATTCTG AATTTGGTCC AATTTATTGG

                      1401                                              1450
GRMZM2G063306_T02orf  GCTCGTGCTG ACCCTTAAA CCATTCCTAG CAGTACCGAA ACACTATAAA.
ZmTOM1pcr
ZmTOM1orf             GCTCGCGCTG ACCCTTAAA CCATTCCTAG CAGTACCGAA ACACTATAAA.
```
